# Two types of peptides derived from the neurotoxin GsMTx4 inhibit a mechanosensitive potassium channel by modifying the mechanogate

**DOI:** 10.1016/j.jbc.2022.102326

**Published:** 2022-08-04

**Authors:** Nan Zhou, Hui Li, Jie Xu, Zhong-Shan Shen, Mingxi Tang, Xiao-Hui Wang, Wan-Xin Su, Masahiro Sokabe, Zhe Zhang, Qiong-Yao Tang

**Affiliations:** 1 Jiangsu Province Key Laboratory of Anesthesiology, Xuzhou Medical University, Xuzhou, Jiangsu Province, China; 2 Department of Anesthesiology, Ninth People’s Hospital of Suzhou, Suzhou, Jiangsu 215200, China; 3 Department of Pathology, The Affiliated Hospital of Southwest Medical University, Luzhou, Sichuan, China; 4 Mechanobiology Laboratory, Nagoya University, Graduate School of Medicine, Nagoya, Japan; 5 Human Systems Information Laboratories, Kanazawa Institute of Technology, Nonoichi, Japan; 6 Jiangsu Province Key Laboratory of Anesthesia and Analgesia Application Technology, Xuzhou Medical University, Xuzhou, Jiangsu Province, China; 7 NMPA Key Laboratory for Research and Evaluation of Narcotic and Psychotropic Drugs, Xuzhou Medical University, Xuzhou, Jiangsu Province, China

**Keywords:** atrial fibrillation, mechanosensitive channel, mechano-specific inhibitor, BK channel, neuropeptide, GsMTx4, patch clamp, single-channel recording, heart, molecular dynamics simulation, AAD, antiarrhythmic drug, AF, atrial fibrillation, BK, big potassium, cDNA, complementary DNA, CHO, Chinese hamster ovary, ICK, inhibitor cystine-knot, MD, molecular dynamics, MS, mechanosensitive, MSC, mechanosensitive channel, mSlo1, mouse Slo1, RCK, regulator of K^+^ conductance, SA, stretch-activated, SAC, stretch-activated channel, SAKcaC, stretch-activated big potassium channel, STREX-del, STREX-deleted mutation, VSTX1, voltage sensor toxin X1

## Abstract

Atrial fibrillation is the most common sustained cardiac arrhythmia in humans. Current atrial fibrillation antiarrhythmic drugs have limited efficacy and carry the risk of ventricular proarrhythmia. GsMTx4, a mechanosensitive channel–selective inhibitor, has been shown to suppress arrhythmias through the inhibition of stretch-activated channels (SACs) in the heart. The cost of synthesizing this peptide is a major obstacle to clinical use. Here, we studied two types of short peptides derived from GsMTx4 for their effects on a stretch-activated big potassium channel (SAKcaC) from the heart. Type I, a 17-residue peptide (referred to as Pept 01), showed comparable efficacy, whereas type II (*i.e.*, Pept 02), a 10-residue peptide, exerted even more potent inhibitory efficacy on SAKcaC compared with GsMTx4. We identified through mutagenesis important sequences required for peptide functions. In addition, molecular dynamics simulations revealed common structural features with a hydrophobic head followed by a positively charged protrusion that may be involved in peptide channel–lipid interactions. Furthermore, we suggest that these short peptides may inhibit SAKcaC through a specific modification to the mechanogate, as the inhibitory effects for both types of peptides were mostly abolished when tested with a mechano-insensitive channel variant (STREX-del) and a nonmechanosensitive big potassium (mouse Slo1) channel. These findings may offer an opportunity for the development of a new class of drugs in the treatment of cardiac arrhythmia generated by excitatory SACs in the heart.

Atrial fibrillation (AF), the most common cardiac arrhythmia, is usually associated with passive stretching in the atrial chamber arising from hemodynamic or mechanical disorders of the heart (*e.g.*, hypertension, mitral valve disease, cardiac failure) (1, 2, 3). Currently approved conventional antiarrhythmic drugs (AADs), including sodium channel blockers and potassium channel blockers, have limited efficacy, especially in persistent AF patients. In addition, these blockers may carry a risk of ventricular proarrhythmia (4, 5). As the excitatory currents carried by stretch-activated (SA) channels (SACs) in the heart have been suggested to generate fast arrhythmias (1, 6, 7), the identification of an effective inhibitor for SACs in the heart may provide an opportunity for developing a new class of antiarrhythmic reagents against the causes rather than the symptoms.

In the heart, atrial dilatation and stretch have been shown to play important roles in the occurrence and maintenance of AF (8, 9). Mechanical stress could increase excitability and initiate arrhythmias and failure through the SAC in the heart (1, 10). Experimental evidence indicated that the excitatory currents carried by SACs can promote fast arrhythmias during the stretch (2, 6). Thus, specific inhibition of SACs in the heart could provide a novel therapy for cardiac arrhythmogenesis (1, 10). SACs are ubiquitous and have been found in cardiac tissues of various species, including humans (11, 12, 13, 14). Gadolinium ion (Gd^3+^), a potent SAC inhibitor, could decrease stretch-induced vulnerability to cardiac fibrillation without influence on the refractory period (1, 2); however, Gd^3+^ also blocks other ion channels, it lacks specificity (15, 16), and thus is not clinically applicable.

Polypeptide toxins have played a central part in understanding the physiological and pathological functions of ion channels (17, 18), which led to important advances in basic research, and even to clinical applications (19, 20, 21). Recently, neuropeptide GsMTx4 extracted from the venom of *tarantula Grammostola spatulata* (22) has been found to inhibit a wide variety of mechanosensitive (MS) channels (MSCs) (23, 24, 25, 26) and has been identified as a selective MSC inhibitor. GsMTx4 inhibits SA currents in the heart and the central nervous system (12, 22, 27, 28). It also was shown to suppress stretch-induced vulnerability to AF without influence on the refractory period and action potential under stretching, making this peptide a potential AAD candidate for the treatment of AF (1). Nevertheless, synthesizing the folded structure with three disulfide bonds limits its application and the development of therapeutic drugs. The primary aim of this study was to identify short peptides that could mimic the function of peptide GsMTx4 on MSCs by using a SA big potassium (BK) channel (SAKcaC) from the heart as a model channel.

Spider toxin GsMTx4 belongs to the inhibitor cystine-knot (ICK) peptide, a common feature for venom toxins (16, 29, 30, 31). It acts as a gating modifier and targets mechanogating (the process of opening and closing) of the MSC by partitioning into the cell membrane, as other ICK peptides do (22, 27, 32). Structural analysis reveals that GsMTx4 is composed of 34 amino acids, the six cysteines in the backbone form three cystine knots and are folded into two doughnut-like interconnected rings (16, 30). Another prominent feature for GsMTx4 is that the structure contains a belt of charged residues surrounding a hydrophobic patch. Sequence comparison between the two mechanotoxins (GsMTx-4 and GsMTx-2) and with other homologous ICK peptides (*e.g.*, hanatoxin, voltage sensor toxin X1, etc.) shows less similarity between the two MS peptides (GsMTx4 *versus* GsMTx2) than among GsMTx4 to other ICK peptides (*e.g.*, GsMTx4 *versus* hanatoxin) (16, 30). Even though, none of these ICK peptides (*e.g.*, hanatoxin, voltage sensor toxin X1, etc.) has been identified as an inhibitor of the MSCs. Thus, the inhibitory action of GsMTx4 on MSCs may be explained by a feature that is not shared with other peptides (16, 30). We noticed that the backbone folds in loop2 and loop3 in the two mechanotoxins (GsMTx4 *versus* GsMTx2) are highly conserved (16). We thus predicted that the regions between loop2 + loop3 in GsMTx4 are responsible for its action on the MSCs. As the backbone in loop2 between the two mechanotoxins (GsMTx4 and GsMTx2) is nearly superimposable, whereas that in loop3 of GsMTx4 shows greater similarity to hanatoxin than to GsMTx2 (16), loop3 does not seem to be essential for the selective action of peptide GsMTx4 on MSCs. We thus predicated that loop2 in GsMTx4 is responsible for its specific inhibition action on the MSCs. In this study, two types of short peptides, one (*e.g.*, Pept 01) derived from the loop2 + loop3 in GsMTx4, and the other (*e.g.*, Pept 02, Pept 03, Pept 04, and Pept 05, etc.) from loop2 were designed; we tested their inhibition efficacy on a SAKcaC as well as the non-MS variant (STREX-del) and a regular BK (mouse Slo1 [mSlo1]) channel.

## Results

### Peptide design strategy

The neuropeptide GsMTx4 has been reported to selectively inhibit MS ion channels (12, 16); the solution structure in water reveals that GsMTx4 contains four loops that are constrained with three cystine knots similar to other ICK toxins from venom (16, 33). To examine whether a short peptide derived from GsMTx4 would function similarly to GsMTx4, we designed and synthesized two types of short peptides based on the similarities among ICK toxins and tested their effects on SAKcaC.

**Figure 1:**
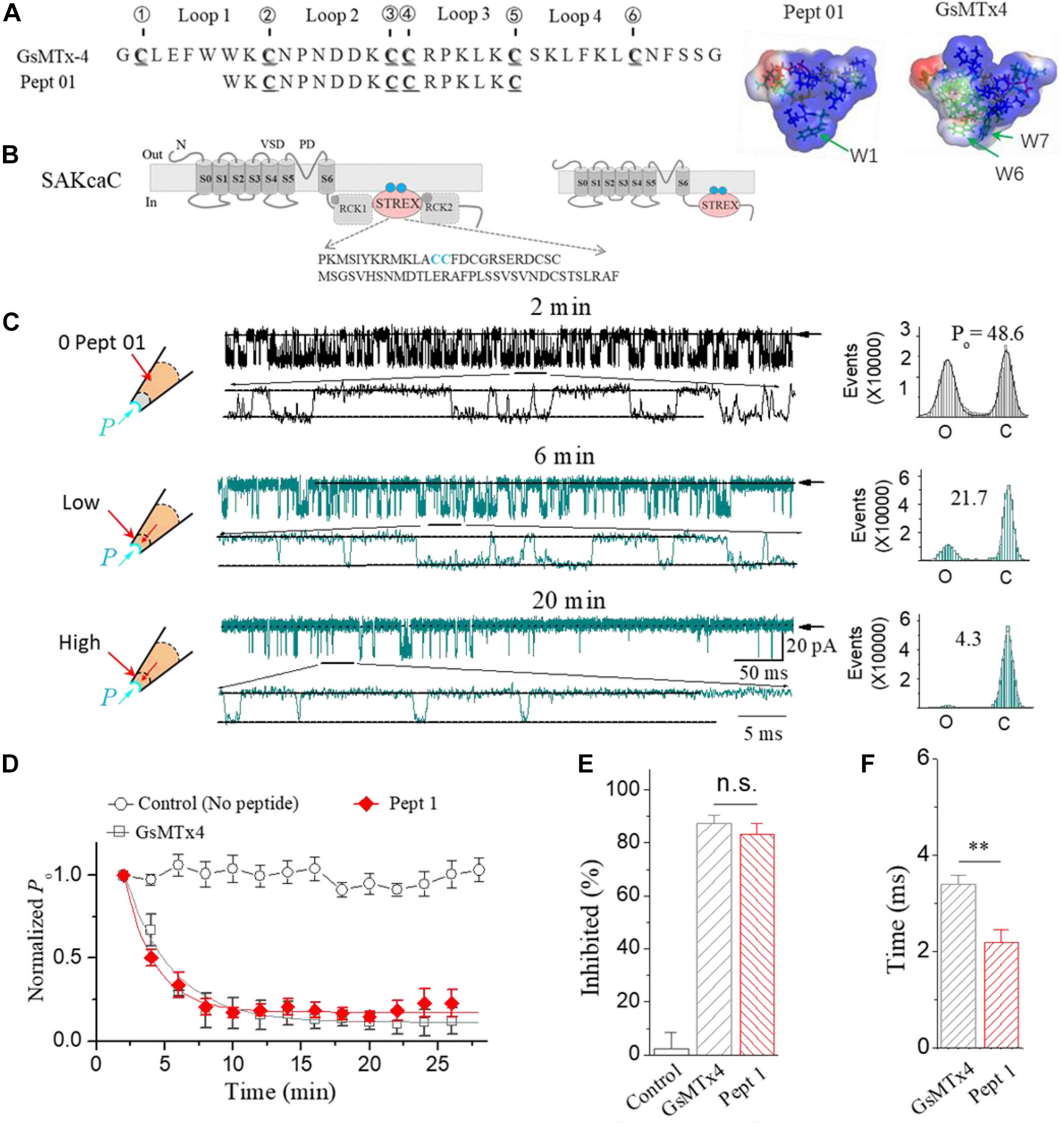
**The synthetic peptide 01 (Pept 01), derived from loop2 and loop3 in neuropeptide GsMTx4, inhibits the stretch-activated BK (SAKca) channel (SAKcaC).***A*, sequence of Pept 01. *B*, schematic illustrates the topology of mechanosensitive BK channel (SAKcaC). The mechanosensory domain, STREX-exon, is located between RCK1 and RCK2 domains in BK C terminus and targets the plasma membrane *via* palmitoylation of two cysteine residues in the STREX-exon (highlighted in *blue spheres*) (39, 40). The *gray spheres* represent the two Ca^2+^-binding sides that are located in the RCK1 and RCK2 domains. *C*, typical current traces showing the inhibition effect of Pept 01 on mechanical-sensitive SAKcaC. The times above indicate the time points upon peptide backfilled in the pipette (see the Experimental procedures section). Membrane potential (*V*_m_) was held at −80 mV. The total histogram events of channels open (O_1_ and O_2_) and closed (C) were fitted by the Gaussian function on the *right*. *D*, time courses of normalized open probability (*P*_o_/*P*_o(control)_) for control (no peptide) and during Pept 01 diffusion to the cell membranes. The effect of the whole length of peptide GsMTx4 was shown for comparison. *E*, bars represent the inhibited effect (inhibited [%]) for Pept 01 on SAKcaC compared with GsMTx4. The inhibited (%) was obtained 20 min later following backfilling. *F*, the inhibition rates (τ) of Pept 01 on the SAKcaC compared with GsMTx4. τ were obtained with the standard single-exponential function fits in *C*. Membrane potentials (*V*_m_) were held at −80 mV. The *insets* beside *A* represent the 3D model of Pept 01 established from the known structure of GsMTx4 (Protein Data Bank code: 1TYK). Time points were measured from the onset of backfilling for peptides in the extracellular side of the cell membrane (see the Experimental procedures section). Peptide concentrations used were 5 μM. The *cartoons* on the *left* in *C* represent the pipette backfilled with Pept 01 with tension (*P*_m_) automatically formed upon membrane deformation following the excised inside–out patch-clamp configuration. The *arrows* beside the traces represent the level of the channel closed. ∗∗*p* < 0.01 *versus* GsMTx4. n *=* 4 to 8. Intracellular calcium ([Ca^2+^]_i_) was 1 mM. SAKcaCs were recorded from the CHO-expressing system. BK, big potassium; CHO, Chinese hamster ovary; ns, not significant; RCK, regulator of K^+^ conductance.

**Figure 2:**
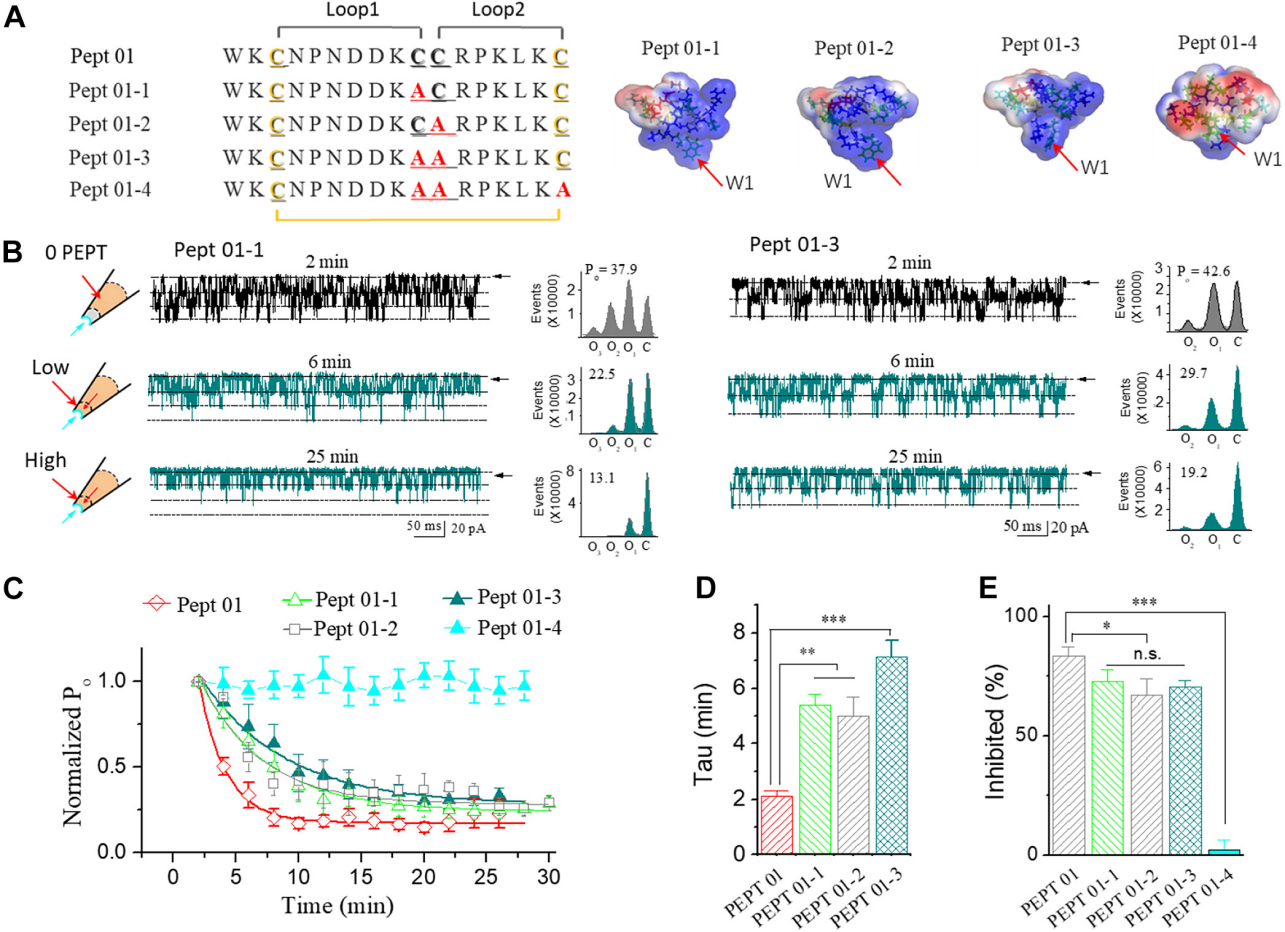
**Cys17 is important for type I (Pept 01) function in the absence of Cys**^**10**^**/Cys**^**11**^**.***A*, sequences for Pept 01 and its mutant peptides (referred to as Pept 01-1, Pept 01-2, Pept 01-3, and Pept 01-4). The *insets* on the *right* show the 3D structural models for the mutant short peptides, which were established with MD simulation technology. *B*, typical current traces showing the effect of Pept 01-1 on the SAKcaC. The times above indicate the time points following the backfilling of peptide in the pipette (see the Experimental procedures section). The *cartoons* on the *left* represent Pept 01-1 backfilled in the pipette with tension (*P*_m_) automatically formed upon membrane deformation following the excised inside–out patch-clamp configuration. The *rights* show the total histogram events of channels open (O_1_, O_2,_ and O_3_) and closed (C) that were fitted by the Gaussian function. *C*, the same as in *B*, but for the effect of Pept 01-3, of which two cystines (Cys^10^/Cys^11^) were substituted with Ala. *D*, time courses of normalized open probability (*P*_o_/*P*_o(control)_) for Pept 01-1, Pept 01-2, Pept 01-3, and Pept 01-4 on SAKcaC during peptide diffusion to the cell membranes. The effect for short Pept 01 on the SAKcaC was shown for comparison. Time points were measured from the onset of backfilling for peptides in the extracellular side of the cell membrane. Peptide concentrations used were 5 μM. *D*, *bars* represent the inhibition rates (τ) for Pept 01-1, Pept 01-2, and Pept 01-3 on the SAKcaC that was compared with Pept 01. τ were obtained with the standard single-exponential function fits in *C*. *E*, the inhibited effects (inhibited [%]) for Pept 01-1, Pept 01-2, Pept 01-3, and Pept 01-4 on SAKcaC. The effect for Pept 01 was shown for comparison. The inhibited (%) was obtained 20 min later following backfilling. Membrane potentials (*V*_m_) were held at −80 mV. ∗∗*p* < 0.01, ∗∗∗*p* < 0.001 *versus* Pept 01. Data points represent at least four experimental determinations. Intracellular calcium ([Ca^2+^]_i_) was 1 mM. SAKcaC was recorded from the CHO-expressing system. CHO, Chinese hamster ovary; MD, molecular dynamics; SAKcaC, stretch-activated big potassium channel.

Theoretically, toxins having similar physiological functions would be expected to share similar sequence and/or structural features (16). Even though, GsMTx4 shares less homology in sequence with GsMTx2, another mechanotoxin (∼11% identity and ∼28% similarity) than to hanatoxin (∼28% identity and ∼37% similarity) (16) (Fig. S1). However, hanatoxin does not inhibit MSCs (16). Likewise, the voltage sensor toxin X1 (VSTX1) that blocks voltage-sensitive ion channels has an even greater identity and similarity to GsMTx4 (∼43.2% identity and ∼54.3% similarity), nevertheless, VSTX1 does not block MSCs (16, 30, 34). These correlations indicated that examining the linear sequence of the full-length peptide may not be sufficient for understanding the efficacy of blocking MSCs. We noticed that the backbone folds in loop2 + loop3 share higher similarity between the two mechanotoxins (16) (*e.g.*, loop2 + loop3 in GsMTx4 shares up to ∼41% identity and ∼53% similarity to GsMTx2 but with a lower identity of ∼35% and a similarity of ∼41% to hanatoxin). These led us to postulate that the functional fragment in GsMTx4 might be among the residues between in loop 2 and loop 3 (as we designed as the type I peptide Pept 01, from Trp^7^ to Cys^17^ in GsMTx4; Figs. 1 and 2).

Likewise, the backbone fold in loop3 of GsMTx4 shows an even higher similarity to hanatoxin than to GsMTx2, but the loop2 between GsMTx4 and GsMTx2 is nearly superimposable (16); these observations allow us to predict that the loop2 in GsMTx4 may be critical for peptide function (as we designed as the type II peptide in this study, *e.g.*, Pept 02, Pept 03, Pept 04, and others, which contains loop2, the hydrophobic residue Trp^7^, and the positively charged residue Lys^8^ in loop1 of GsMTx4 (Fig. 4).

**Figure 3:**
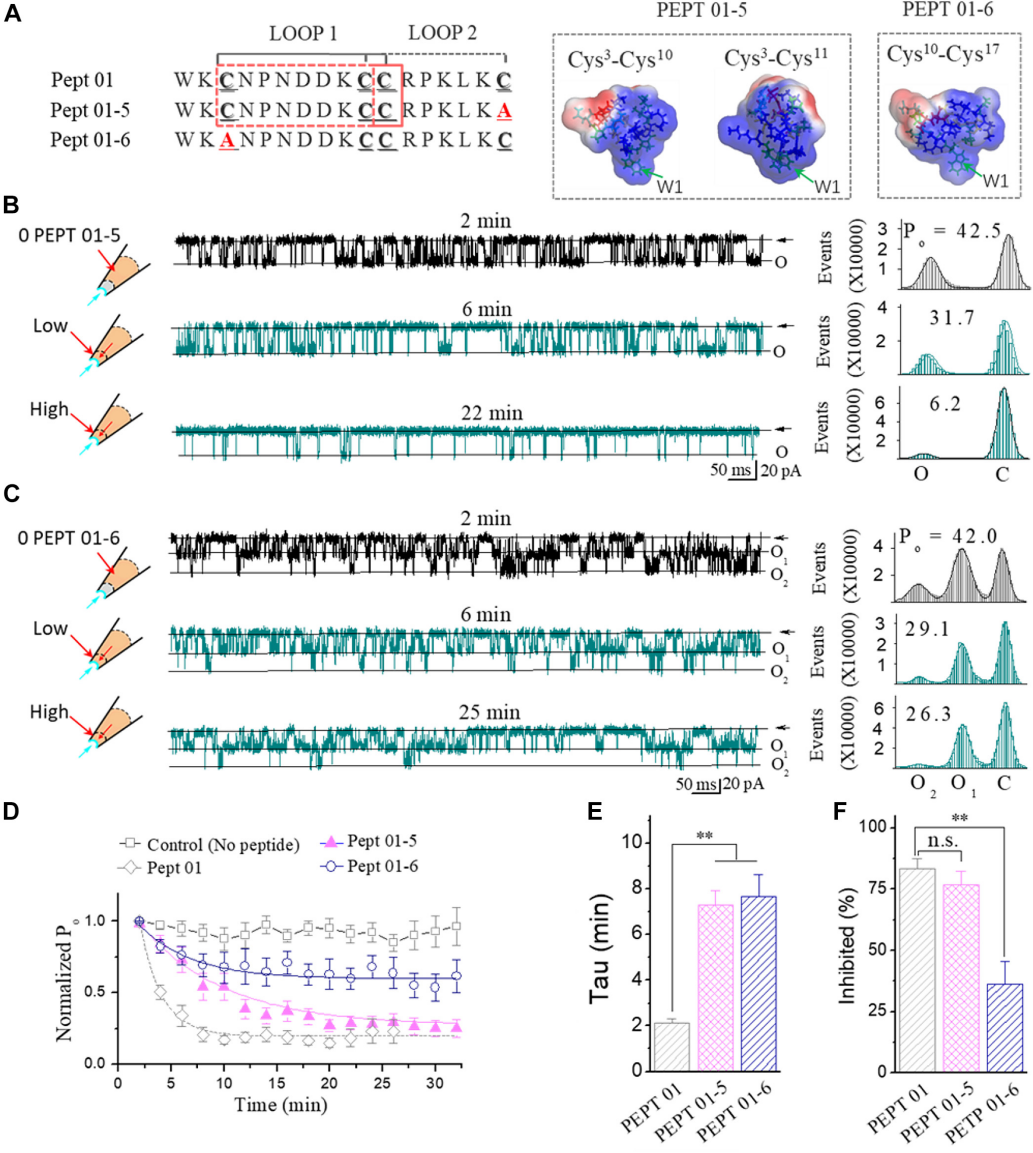
**Cys3 is essential for type I (Pept 01) function.***A*, sequences for Pept 01-5 and Pept 01-6, of which Cys^17^ was substituted with Ala in Pept 01 for Pept 01-5, and Cys^3^ substituted for Pept 01-6. The *insets* on the *right* show the 3D structural models for the short Pept 01-5 and Pept 01-6 that were established with MD simulations. Note, Pept 01-5 folds in two states (Fig. S4). *B*, typical single-channel traces showing the effect of Pept 01-5 on the SAKcaC. The times above indicate the time points following the backfilling of peptide in the pipette (see the Experimental procedures section). The *rights* show the total histogram events of channels open (O) and closed (C) that were fitted by the Gaussian function. *C*, the same as in *B*, but for the effect of Pept 01-6, of which Cys^3^ in Pept 01 was substituted with Ala. *D*, time courses of normalized open probability (*P*_o_/*P*_o(control)_) for Pept 01-5 and Pept 01-6 during peptide diffusion to the cell membranes. The effect of the short peptide Pept 01 on the SAKcaC was shown for comparison. Time points were measured from the onset of backfilling for peptides in the extracellular side of the cell membrane. Peptide concentrations used were 5 μM. *E*, *bars* represent the inhibition rates (τ) on the SAKcaC for Pept 01-5 and Pept 01-6 compared with Pept 01. τ were obtained with the standard single-exponential function fits in *D*. *F*, the inhibited effects (inhibited [%]) for Pept 01-6 and Pept 01-6 on SAKcaC. The effect for Pept 01 was shown for comparison. The inhibited (%) was obtained 20 min later following backfilling. Membrane potentials (*V*_m_) were held at −80 mV. ∗∗*p* < 0.01, ∗∗∗*p* < 0.001 *versus* Pept 01. Data points represent at least four experimental determinations. Intracellular calcium ([Ca^2+^]_i_) was 1 mM. SAKcaC was recorded from the CHO-expressing system. CHO, Chinese hamster ovary; MD, molecular dynamics; SAKcaC, stretch-activated big potassium channel.

**Figure 4:**
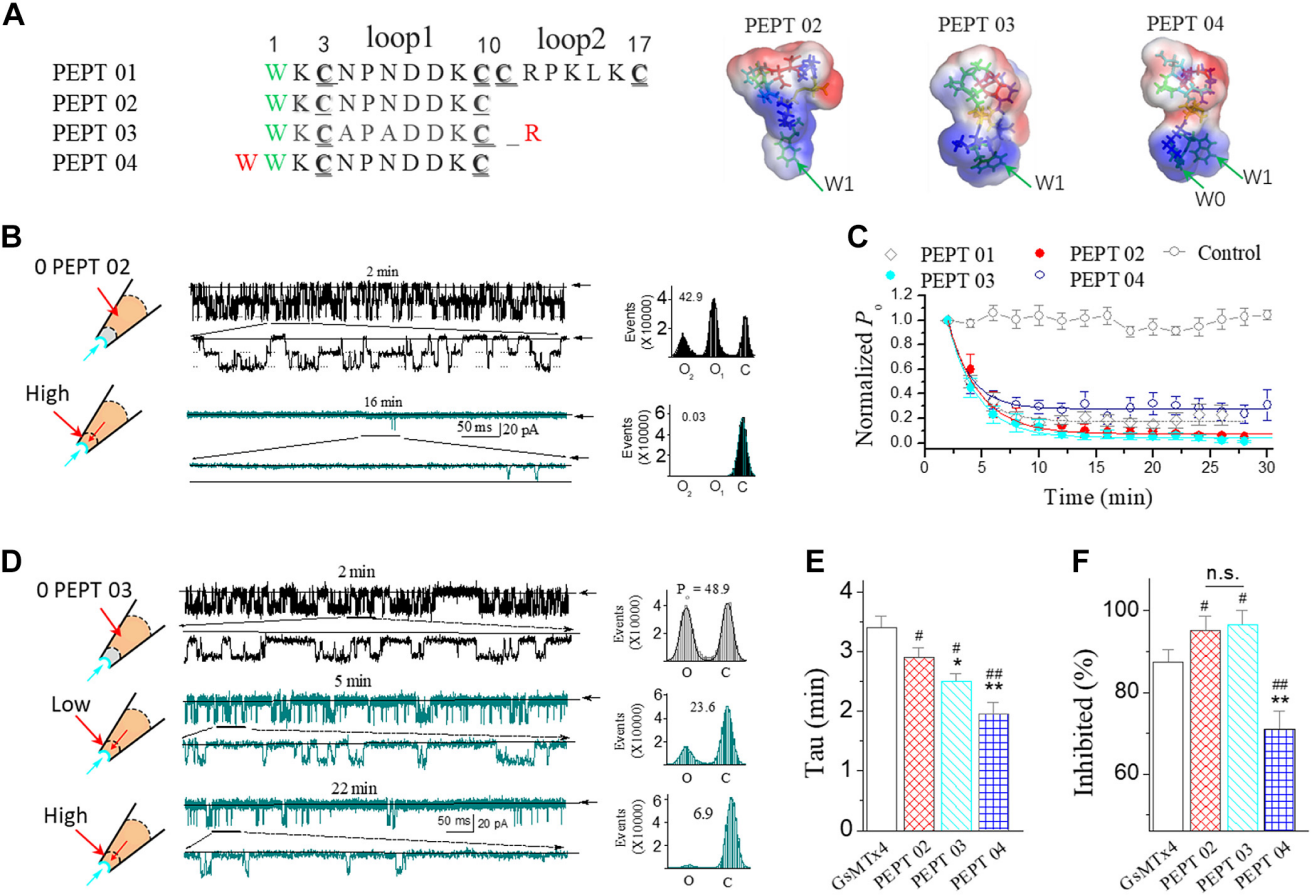
**The synthetic peptides (type II) derived from loop2 of GsMTx4 sustains the essential inhibitory effect of GsMTx4 on the stretch-activated BK channel.***A*, sequences for peptides designed from loop2 of GsMTx4 (*e.g.*, Pept 02, Pept 03, and Pept 04). The *insets* on the *right* show the surface images of Pept 02 and Pept 03. Hydrophobic residues (Ala, Cys, Ile, Leu, Met, Phe, Pro, Trp, Tyr, and Val) are in *green*, positive residues (Arg and Lys) are in *blue*, and negative residues (Asp and Glu) are in *red*. *B*, the sample traces showing the inhibition effect of Pept 02 on the mechanosensitive SAKcaC. The *rights* show the corresponding histogram events of channels open (O) and closed (C), which were fitted with the Gaussian function. *C*, time courses of normalized open probability (*P*_o_/*P*_o(control)_) for the synthetic Pept 02, Pept 03, and Pept 04 during peptide diffusion to the cell membranes. The control (no peptide) and the effect of Pept 01 were shown for comparison. *P*_o_ was normalized to the control level (*P*_o_/*P*_o(control)_) following the excised inside–out patch configuration (before peptide diffusion to the cell membrane). *D*, the same as in *B*, but for the inhibition effect of Pept 03. *E*, *bars* represent the inhibition rates (τ) for Pept 02, Pept 03, and Pept 04 on the SAKcaC. The result for the full length of GsMTx4 was shown for comparison. Taus were obtained with the standard single-exponential function in *D*. *F*, the inhibited effects (inhibited [%]) for Pept 02, Pept 03, and Pept 04 on SAKCaC. The effect of GsMTx4 was shown for comparison. The *cartoons* on the *left* of *B* and *C* represent the peptide (Pept 02 in *B* and Pept 03 in *C*) backfilled in the pipette with tension (*P*_m_) automatically formed upon membrane deformation following the excised inside–out patch-clamp configuration. Time points were measured from the onset of backfilling for peptides in the extracellular side of the cell membrane (see the Experimental procedures section). Peptide concentrations used were 5 μM. Membrane potentials (*V*_m_) were held at −80 mV. ∗*p* < 0.05, ∗∗*p* < 0.01 *versus* Pept 02; #*p* < 0.05, ##*p* < 0.01 *versus* GsMTx4. n = 4 to 9 per group. Intracellular calcium ([Ca^2+^]_i_) was 1 mM. SAKcaC was recorded from the CHO-expressing system. BK, big potassium; CHO, Chinese hamster ovary; ns not significantly different; SAKcaC, stretch-activated big potassium channel.

**Figure 5:**
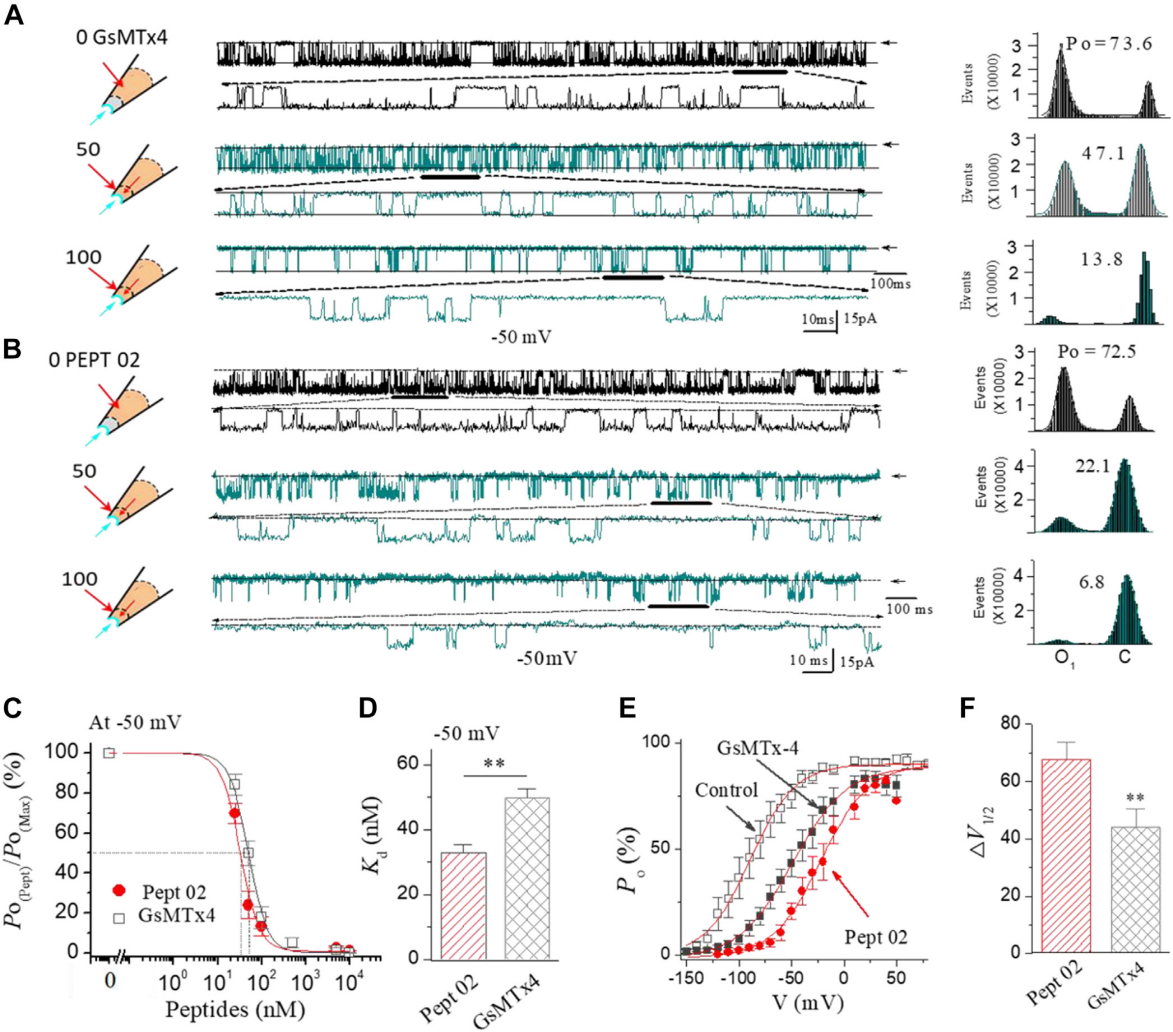
**The synthetic Pept 02 inhibits SAKcaC activity in a dose-dependent manner.***A* and *B*, the sample traces showing the dose-dependent inhibition effects for GsMTx4 (*A*) and Pept 02 (*B*) on the SAKcaC with 0 nM (control, *upper panel*), 50 nM (*middle panel*), and 100 nM (*lower panel*) peptides applied from the extracellular side of the cell membrane. The corresponding total histogram events of channel open (O) and closed (C) states were presented on the *right*. Membrane potential (*V*_m_) was held at −50 mV. Traces were obtained 25 min following the backfilling. *C*, dose–response curves for Pept 02 and GsMTx4 at −50 mV. The *solid lines* are fit to Hill equation with disassociation constants (*K*_*d*_) summarized in *G*. Note, *Y*-axis represents the normalized *P*_o_ (*P*_o(Pept)_/*P*_o(maxi)_). Hill cofactors were 1.8 ± 0.52 for Pept 02 and 1.6 ± 0.47 for GsMTx4. *D*, statistical comparison of the *K*_*d*_ between Pept 02 and GsMTx4 determined from the Hill equation fits in *C*. Data points at each concentration represent three to eight determinations. *E*, *P*_o_–*V* relationships for the effects of Pept 02 and GsMTx4 on the SAKcaC. The peptide concentrations used were 50 nM. The *solid lines* are fits to the standard Boltzmann function: *P*_o_ = *P*_o(max)_/[1 + exp(−(*V*_*m*_ − *V*_1⁄2_)/*K*)], where *V*_1⁄2_ represents the voltage required for half of the maximum channel opening and *K* represents the slope factor. The *V*_1⁄2_ and *K*^−1^ obtained were −91.5 ± 1.36 mV and 25.1 ±1.16 for control (no peptide), −23.7 ± 1.06 mV and 22.9 ± 1.11 for Pept 02, and −47.2 ± 0.97 mV and 24.5 ± 1.63 for GsMTx4. *F*, statistical comparison of △*V*_1/2_ shifted by Pept 02 (*red*) *versus* GsMTx4 (*gray*). *V*_1/2_ was obtained from *E*. △*V*_1/2_ = *V*_1/2(Pept)_ − *V*_1/2(Control)_. n = 6 to 9 per group. ∗∗*p <* 0.01. SAKcaCs were recorded from ventricular myocytes. SAKcaC, stretch-activated big potassium channel.

**Figure 6:**
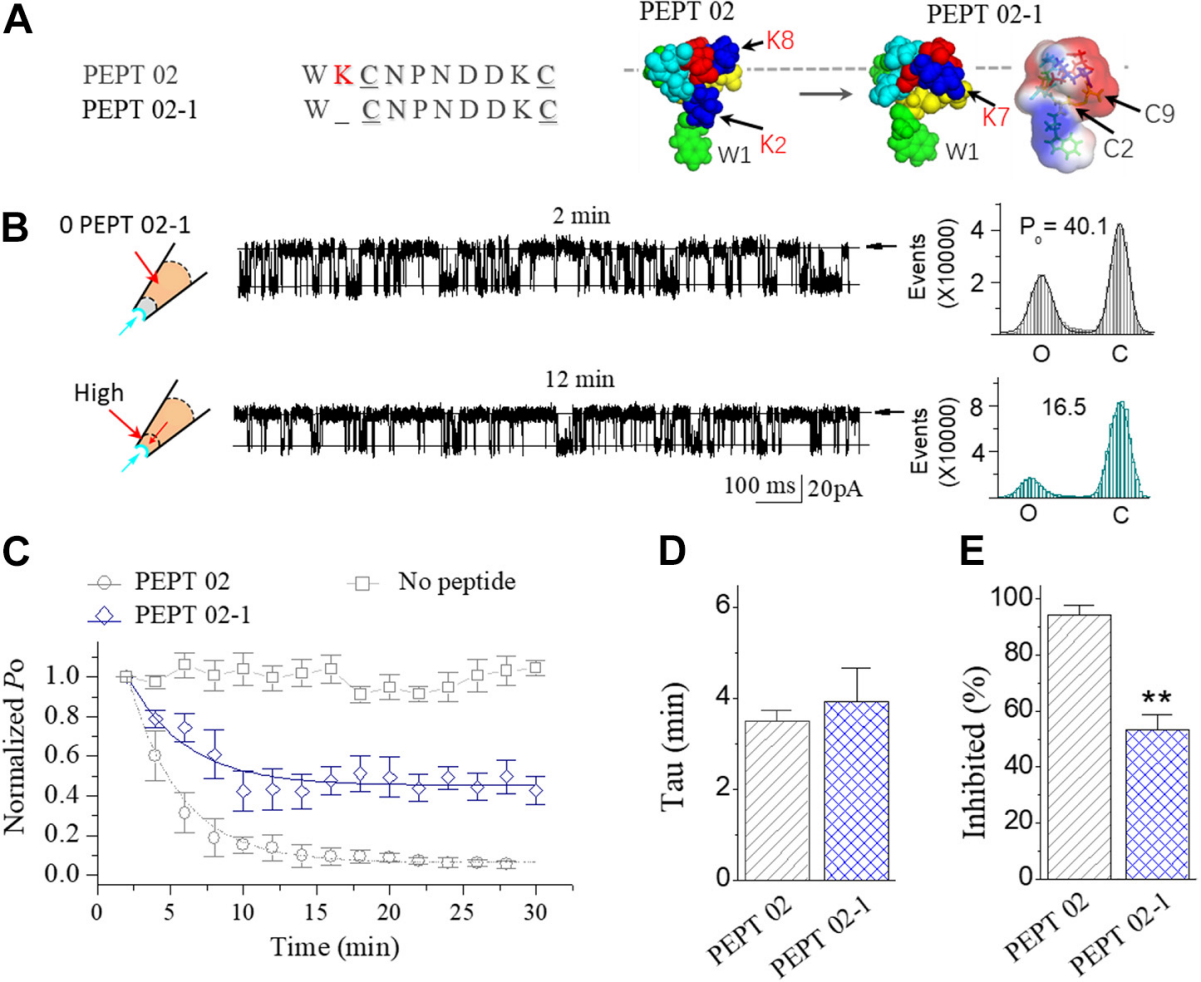
**The positively charged residue of Cys**^**2**^**in short Pept 02 is important for the inhibitory effect of Pept 02 on the stretch-activated BK channel.***A*, sequence for Pept 02-1. The *inset* on the *right* shows the surface image of Pept 02-1, which was obtained from the structure model of Pept 02. *B*, typical traces showing the inhibition effect of Pept 02-1 on the SAKcaC at the time points following backfilling as indicated. The *cartoons* on the *left* represent Pept 02-1 backfilled in pipettes with tension (*P*_m_) automatically formed upon membrane deformation following the excised inside–out patch-clamp configuration. Time points were measured from the onset of backfilling for peptides in the extracellular side of the cell membrane. The corresponding total histogram events of channel open (O) and closed (C) states were shown on the *right*. Each number in the *y*-axis is timed 10,000. Membrane potential (*V*_m_) was held at −80 mV. *C*, time course of normalized *P*_o_ (*P*_o_/*P*_o(control)_) for Pept 02-1 during peptide diffusion to the cell membrane following backfilling at −80 mV (n = 5). Control (no peptide backfilled) and the effect of Pept 02 and control (no peptide) were shown for comparison. *P*_o_ was normalized to the levels following the excised inside–out patch configuration (before peptide diffusion to the cell membrane). *D*, comparison of the inhibited effects (inhibited [%]) between Pept 02-1 and Pep 02. Peptide concentrations used were 5 μM. Data points represent the mean ± SE (error bars) of at least four experiments. ∗∗*p* < 0.005. Intracellular calcium ([Ca^2+^]_i_) was 1 mM. SAKcaC was recorded from the CHO-expressing system. BK, big potassium; CHO, Chinese hamster ovary; SAKcaC, stretch-activated big potassium channel.

**Figure 7:**
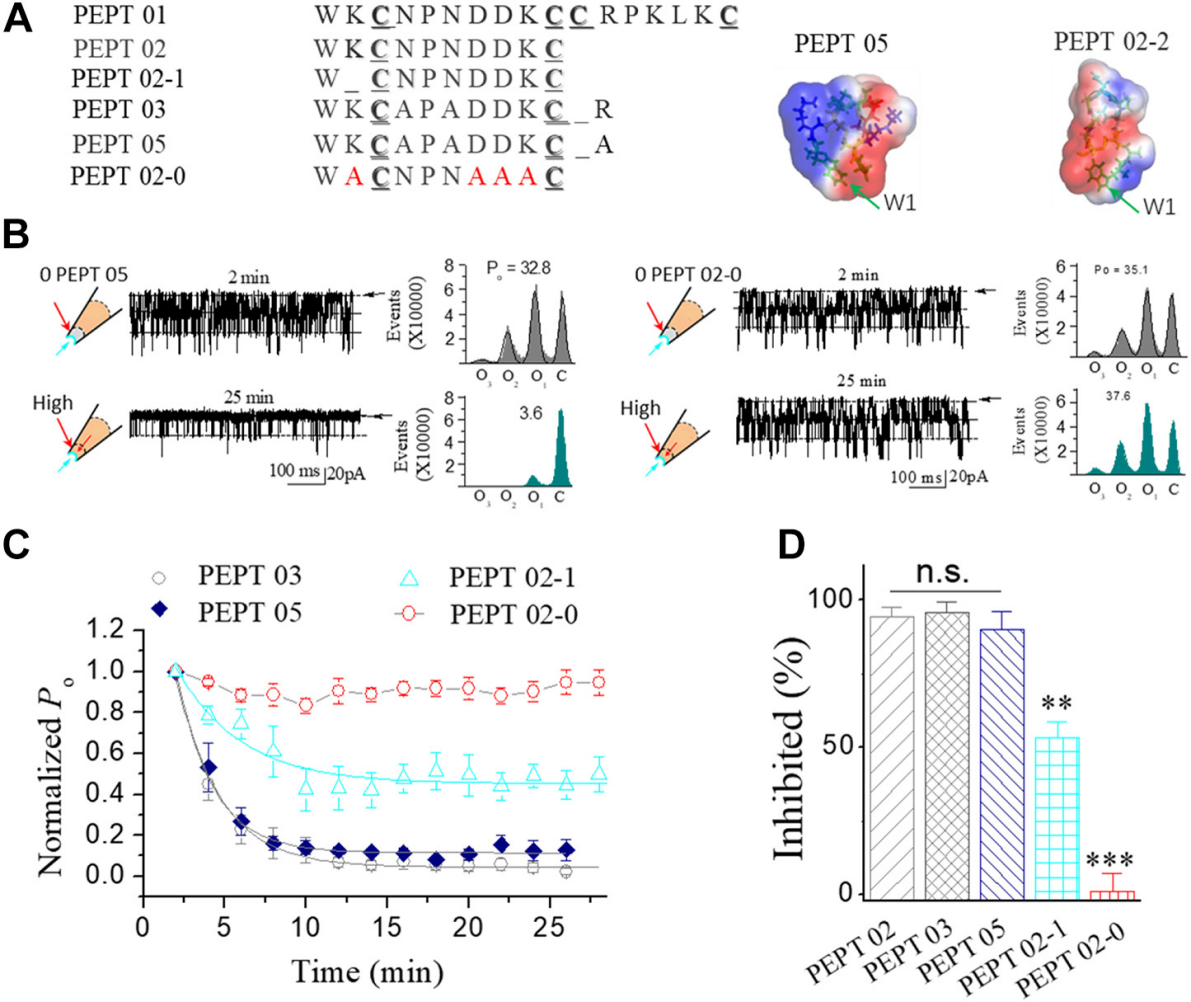
**Neutralization of the charged residues abolishes the inhibitory effects of Pept 02 and Pept 03 on stretch-activated BK channels.***A*, sequences for the synthetic Pept 05 and Pept 02-0, of which all charged residues (Lys 2, Asp7, Asp8, Lys 9, and Arg11) in Pept 05 were neutralized to Ala (as referred to as Pept 02-0). The sequence for Pept 02-1 was shown for comparison. The *insets* on the *right* show the surface images of Pept 05 and Pept 02-0. *B*, the sample traces showing the inhibition effect of Pept 05 (*left*) and Pept 02-0 (*right*) on the SAKcaC obtained at the time point indicated following backfilling. The *cartoons* on the *left* of recording traces represent the Pept 05 (*left*) and Pept 02-0 (*right*) backfilled in pipettes with tension (*P*_m_) automatically formed following the excised inside–out patch-clamp configuration. Time points were measured from the onset of backfilling for peptides in the extracellular side of the cell membrane. The corresponding total histogram events of channel open (O_1_, O_2_, and O_3_) and closed (C) states shown on the *right* were fitted to Gaussian functions. Each number in the *y*-axis is timed 10,000. Membrane potentials (*V*_m_) were held at −80 mV. *C*, time courses of normalized open probability (*P*_o_/*P*_o(control)_) for the synthetic Pept 05 and Pept 02-0 during peptide diffusion to the cell membranes following backfilling. *V*_m_ = −80 mV. The effect of Pept 03 was shown for comparison. *D*, comparison of the inhibited effects (inhibited [%]) among Pept 03, Pept 05, and Pept 02-0. The results for Pept 02-1 were shown (in *C* and *D*) for comparison. Peptide concentrations used were 5 μM. Data points represent the mean ± SE (error bars) of at least four experiments. ∗∗∗*p* < 0.001. Intracellular calcium ([Ca^2+^]_i_) was 1 mM. SAKcaC was recorded from the CHO-expressing system. BK, big potassium; CHO, Chinese hamster ovary; SAKcaC, stretch-activated big potassium channel.

### The effect of type I derived from loop2 + loop3 in GsMTx4 on SAKcaCs

Theoretically, the truncated type I peptide (*e.g.*, Pept 01), derived from Cys^3^ to Cys^17^ of GsMTx4, can form five different forms in solution with different disulfide bonds. The structural model of Pept 01 obtained with molecular dynamics (MD) simulation with the free-product run method (see the Experimental procedures section) presents a concave surface that was commonly found in neurotoxins and possesses a shape similar to GsMTx4 (Fig. 1, the *insets*, also see Discussion section). We first examined whether this short peptide would function similarly as GsMTx4 on the SAKcaC.

We have previously shown that the neuropeptide GsMTx4 inhibited SAKca from chick heart (12), thus we examined the effect of Pept 01 on the SAKcaC and compared it with the blocking effect of GsMTx4 on SAKcaC when expressed in Chinese hamster ovary (CHO)-K1. One unique characteristic of the SAKcaC is that the SAKca gene contains an extra exon (STREX) located between regulator of K^+^ conductance 1 (RCK1) and RCK2 domains in the C terminus (Fig. 1*B*) when compared with the regular BK channel (35, 36). STREX-exon has been identified as one of the mechanosensor domains in the SAKcaC (37, 38). Interestingly, the cytosolic C terminus of the STREX BK channel uniquely interacts with the plasma membrane *via* palmitoylation of two cysteine residues in the STREX domain (39, 40). As the cell membrane can generate forces automatically following the formulation of excised inside–out patch clamp (12, 41), we tested peptide effects on SAKcaC under conditions without additional membrane stretch applied to the cell membrane (12, 41). The synthetic short peptide 1 (Pept 01) was applied from the extracellular side of the cell membrane (in the pipette) by using the standard back-filling method (see the Experimental procedures section), which allows the drug to be gradually dispersed to the outer surface of the ion channel protein in the cell membrane (12, 42, 43).

Similar to the blocking effect of the GsMTx4 on SAKcaC (12), Pept 01 (5 μM) backfilled in the pipette significantly decreased single-channel open probability (*P*_o_) in a time-dependent manner, for example, channel *P*_o_ was decreased from 48.6% to 21.7% at 6 min and further decreased to 4.3% at 20 min following back-filling of the peptide in the pipette (Fig. 1*C*, *right*). Consistent with the previous results (12), we did not observe significant inactivation or rundown for the SAKcaC activity during recordings but with ongoing spontaneous activity in the presence of 1 mM Ca^2+^ in the bath solution (the intracellular side of the ion channel). Time courses for the effect of Pept 01 on SAKcaC activities from five independent recordings were summarized in Figure 1*D*. The gradual decreases in *P*_o_ for the SAKcaC observed in the time-course recordings reflect the gradual diffusion of the peptide onto the surface of the cell membrane (42, 43). Similar to the GsMTx4 effect on the SAKcaC, the single-channel conductance and ion selectivity (K^+^/Na^+^) were not altered by Pept 01 (Fig. S2), suggesting that Pept 01 acts as a gating modifier on SAKcaC as the effect of the parent peptide GsMTx4 on SAKcaC (12).

Although we did not observe a significant difference in the inhibition effects between the short Pept 01 and GsMTx4 under the same conditions (Fig. 1*E*), the averaged diffusion/inhibitory rate (τ) of Pept 01 on the SAKcaC was significantly smaller than that of GsMTx4 (Fig. 1*F*), suggesting the increased diffusion rate of the peptide partitioning into the cell membrane and/or the enhanced peptide channel–lipid interaction. We suppose that the faster diffusion rate might arise from the smaller/sharper hydrophobic protrusion (formed with one tryptophan [Trp1] at the head of Pept 01), whereas two tryptophans (Trp6/Trp7) for GsMTx4 were functionally located at the corresponding location (also see Discussion section).

### Cysteines are important for Pept 01 action on SAKcaC

#### Cys^17^ is important for type I (Pept 01) peptide function when both cysteines of Cys^10^/Cys^11^ were substituted

Given the effective inhibitory effect of Pept 01 on the mechanical-sensitive SAKcaC, we sought to understand the structural components of the peptide that may be critical for its function on SAKcaC. Structurally, toxin GsMTx4 itself is a small amphipathic molecule with an ICK. The three disulfide bonds formed with six cysteines act to constrain the 3D structure of peptides for function (16, 29). To investigate the functional role of cysteines in Pept 01, we first designed three mutant peptides to study the functional role of two cysteines (Cys^10^/Cys^11^): two mutations with one cysteine (Cys^10^ or Cys^11^) substituted to Ala (referred to as Pept 01-1, Pept 01-2, Fig. 2*A*), and the other with both to Ala (referred to as Pept 01-3, Fig. 2*A*). We then examined the effects of the designed peptides on the SAKcaC under the same condition.

As shown in Figure 2*B* (*left*), *P*_o_ of SAKcaC was significantly reduced by Pept 01-1 (5 μM) when backfilled in the pipette, for example, *P*_o_ was significantly decreased from 37.9% to 22.5% at 6 min and further decreased to 13.1% at 25 min following the backfilling for this particular patch recording. The averaged diffusion/inhibitory rate (τ) of Pept 01-1 on the SAKcaC was significantly slowed down (Fig. 2, *C* and *D*). The slowed inhibition rate (τ) may reflect the decreased partitioning rate for Pept 01-1 when compared with Pept 01. The average inhibited (%) by Pept 01-1 showed a significantly decreased efficacy on SAKcaC when compared with Pept 01-1 (Fig. 2, *C* and *E*), suggesting a weakened efficacy of Pept 01-1 on SAKcaC. We did not observe a significant difference between Pept 01-1 and Pept 01-2 for the inhibition of SAKcaC (Fig. 2, *C*–*E*). In addition, when both cysteines of Cys10/Cys11 were mutated to Ala (referred to as Pept 01-3), SAKcaC *P*_o_ was partially reduced by Pept 01-3 (5 μM) when backfilled in the pipette, for example, *P*_o_ was significantly decreased from 42.6% to 29.7% at 6 min but sustained at a relatively higher opening level even at 25 min following the backfilling for this particular patch recording (*P*_o_ = 19.2%), indicating the reduced inhibitory effect of Pept 01-3 when compared with Pept 01. The averaged diffusion/inhibitory rate (τ) of Pept 01-3 on the SAKcaC was significantly slowed down when compared with Pept 01 (Fig. 2, *C* and *D*).

Notably, there were no significant differences in either the diffusion/inhibitory rate (τ, Fig. 2*D*) or the inhibited (%, Fig. 2*E*) for three mutant peptides (Pept 01-1/Pept 01-2/Pept 01-3) on SAKcaC, suggesting that both cystines (Cys^10^/Cys^11^) may play a lower contribution on type I peptide function. Nevertheless, the further substitution of Cys^17^ with Ala in Pept 01-3 (referred to as Pept 01-4, Fig. 2*A*) eliminated the efficacy of Pept 01-3 on SAKcaC (Fig. 2, *C* and *E*), suggesting that Cys^17^ may be required for function when Cys^10^ and Cys^11^ are mutated or that at least one disulfide bridge (Cys^3^-Cys^10^ or Cys^3^-Cys^11^) is required for peptide stability.

Structural analysis obtained with MD simulation for type I and the mutant peptides (Pept 01/Pept 01-1/Pept 01-2/Pept 01-3) revealed that regardless of whether there is one (*e.g.*, Pept 01-1, Pept 01-2) or two (*e.g.*, Pept 01) or the absence (*e.g.*, Pept 01-3) of the cysteines (Cys^10^/Cys^11^), the pattern of Cys^3^-Cys^17^ forming a disulfide bond shares in four peptides (Fig. S3, *A* and *B*). The disulfide bond in ICK peptides has been suggested to act to stabilize and/or facilitate the folding structures (16). Interestingly, when Cys^17^ in Pept 01-2 is further substituted, Pept 01-3 structure becomes disordered (Fig. S3*C*), which does not look like having the potency to partition into the cell membrane, as the hydrophobic protrusion formed with Trp^1^ is folded inside the spatial 3D structure. On the other hand, as there is one cysteine (Cys^3^) left in Pept 01-4, the peptide may not be able to be constrained properly for functioning. The structural model for Pept 01-4 supports the result that Pept 01-4 eliminates the inhibitory effect of Pept 01 on SAKcaC (also see the Discussion section).

#### Cys^3^ is essential for type I (Pept 01) peptide function on MS BK channel

We next investigated how the individual cysteine Cys^3^/Cys^17^ affects the function of type I (Pept 01) peptide on SAKcaC. For this purpose, we designed two mutant peptides, one with Cys^17^ substituted to Ala (referred to as Pept 01-5, Fig. 3*A*) and the other with Cys^3^ to Ala (referred to as Pept 01-6, Fig. 3*A*) and then examined the inhibitory effects on the SAKcaC under the same conditions.

As shown in Figure 3*B*, *P*_o_ of the SAKcaC was significantly inhibited by Pept 01-5 (5 μM). It reduced from 42.5% to 31.7% at 6 min and to 6.2% at 22 min following the backfilling. Interestingly, under the same conditions, *P*_o_ sustained at a relatively higher opening level even at 25 min (*P*_o_ = 26.3%) following the backfilling of Pept 01-6 (Fig. 3*C*), indicating a significantly weakened inhibitory effect for Pept 01-6 when compared with Pept 01-5 (*P*_o_ was decreased ∼37.4% by Pept 01-6 *versus* ∼76.0% by Pept 01-5, Fig. 3, *B*–*D*). Although the averaged diffusion/inhibitory rate (τ) for both Pept 01-5 and Pept 01-6 on SAKcaC were dramatically slowed down when compared with Pept 01 (Fig. 3*D*), for example, τ was 7.3 ± 0.6 min for Pept 01-5, 7.6 ± 0.9 for Pept 01-6, and 2.1 ± 0.2 for Pept 01 (Fig. 3*E*), the inhibition (%) for Pept 01-5 on SAKcaC was comparable with that of Pept 01, whereas the inhibition (%) by Pept 01-6 on SAKcaC was mostly abolished (Fig. 3*F*). We concluded that Cys^3^ is essential for Pept 01 function. MD simulation results indicated that in the absence of Cys^17^, Cys^3^ in Pept 01-5 can form a disulfide bond with Cys^10^ or Cys^11^ (folded to loop1) (Fig. S4), whereas when Cys^3^ was substituted, the pair of Cys^10^-Cys^17^ forms a disulfide bond and folded to a loop (loop2). As Pept 01-5 shows a comparable inhibitory efficacy with type I (Pept 01) peptide, it is most likely that loop1 in Pept 01 is the functional loop for type I (Pept 01) (see Discussion section).

### The effect of peptides derived from loop2 in GsMTx4

As the backbone fold in loop2 of GsMTx4 is nearly superimposable with that in GsMTx2, another MS ion channel–selective inhibitor (16), we next examined whether the short peptides, derived from loop2 of GsMTx4, could still function on the SAKcaC. We thus created a truncated peptide from the loop2 of GsMTx4 (corresponding to loop 1 in Pept 01, referred to as Pept 02, Fig. 4*A*) and tested its effect on SAKcaC. As shown in Figure 4*B*, the same concentration of Pept 02 (5 μM) significantly decreased SAKcaC *P*_o_ when backfilled in the pipette. For example, the *P*_o_ of the SAKcaC was significantly decreased from 42.9% to 3.6% at 16 min following backfilling for this particular recording. The averaged *P*_o_ was decreased gradually with the time following backfilling (*red-filled circles*, Fig. 4*C*), which reflects the gradual dispersion of peptide to the outer surface of the ion channel protein in the cell membrane. Pept 02 showed a faster inhibition rate (Fig. 4*E*) and an enhanced inhibition (%) (Fig. 4*F*) on SAKcaC when compared with GsMTx4, suggesting that Pept 02 may mimic GsMTx4 action but act as an even stronger inhibitor on SAKcaC when compared with the parent peptide GsMTx4.

To explore whether one sequence extension from the corresponding positions within GsMTx4 could enhance/affect Pept 02 function, we designed two peptides, one (named Pept 03) contains an additional Arg (Arg^11^) at the ending of Pept 2 (corresponding to Arg^18^ in the loop3 of GsMTx4, Fig. 4*A*), and the other, named as Pept 04, contains an additional Trp (Trp^0^) at the beginning of Pept 02 (corresponding to Trp^6^ in the loop1 of GsMTx4, Fig. 4*A*), We then compared their efficacies on SAKcaC with that of Pept 02. Figure 3*D* showed that the synthetic peptide of Pept 03 did not further enhance the inhibitory effect of Pept 02 on SAKcaC: the same concentration of Pept 03 (5 μM) decreased SAKcaC *P*_o_ from 48.9% to 6.9% at 22 min following backfilling (Fig. 4*D*). As in the case of Pept 02, Pept 03 inhibited SAKcaC in a time-dependent way (Fig. 4*C*). Although the averaged inhibition rate (τ) for Pept 03 was modestly accelerated when compared with Pept 02 (Fig. 4, *C* and *E*), the inhibited (%) effect by Pept 03 was comparable with that by Pept 02 (Figs. 4*C* and 3*F*). We thus concluded that one sequence extension, either at the ending or at the beginning of the peptide, did not further facilitate short peptide function on SAKcaC. The faster inhibition rate for Pept 03, when compared with Pept 02 (Fig. 4*E*), might arise from the stronger electrostatic interaction between the positively charged protrusion and the negatively charged carbonyl oxygen atoms in the inner monolayer (Lys^2^/Arg^11^ in Pept 03 *versus* Lys^2^ in Pept 02, also see Discussion section).

It also has been suggested that the hydrophobic residues in ICK peptide can form the hydrophobic protrusion on the surface of peptide structure, which allows the peptide to penetrate into the cell membrane and interact with the channel/lipid bilayer for further function (27, 31, 44). With the MD simulation method, the tryptophan (Trp^6^/Trp^7^) in GsMTx4 has been suggested to be involved in peptide channel–lipid interaction (12, 27). With one sequence extension at the beginning of Pept 02 (numbered as Trp^0^, corresponding to Trp^6^ in GsMTx4, referred to as Pept 04), and examined its efficacy on SAKcaC, the averaged inhibition rate (τ) for Pept 04 was modestly decreased (the averaged τ was 1.95 ± 0.18 min for Pept 04 *versus* 2.89 ± 0.16 min for Pept 02, Fig. 4*E*); nevertheless, the inhibited (%) by Pept 04 on SAKcaC was significantly decreased when compared with that of Pept 02 (*e.g.*, the inhibited [%] by Pept 04 was 70.9 ± 4.5% *versus* 94.2 ± 3.5% by Pept 02, Figure 4*F*). We concluded that, although an additional Trp^0^ immediately preceding Trp^1^ in Pept 02 modestly hastened the inhibition/absorption rate, the inhibitory efficacy by Pept 04 was significantly weakened when compared with Pept 02 (Fig. 4*F*). It is possible, as two tryptophans at the head of a peptide structure form a bigger hydrophobic protrusion, which might act to reduce/prevent the insertion/absorption of the peptide into the lipid bilayer (see Discussion section).

Taken together, these results suggested that type II peptides derived from loop2 of GsMTx4 (*e.g.*, Pept 02, Pept 03, and Pept 04) may act as potential inhibitors on SAKcaC in a way as the parent peptide GsMTx4 does. Among them, Pept 02 (Trp-Lys-Cys-Asn-Pro-Asn-Asp-Asp-Lys-Cys) may act as the shortest one that keeps the most potent inhibitory effect on SAKcaC.

### The shortest synthetic Pept 02 inhibits SAKcaC in dose- and voltage-dependent manners

We next investigated the dose-dependent effect of Pept 02, the shortest synthetic peptide identified in this study, on SAKcaC, and compared its efficacy with the natural toxin GsMTx4. Consistent with the previous report that the parent peptide GsMTx4 inhibits SAKcaC in a dose-dependent way (12). For example, at *V*_m_ = −50 mV, SAKcaC showed a high level of activity and high frequencies of channel opening and closing for control (without peptide) under the condition of 1 mM Ca^2+^ in the bath (the intracellular side of the cell membrane, Fig. 5*A*, *upper*). Following the application of 50 or 100 nM GsMTx4 from the extracellular side of the cell membrane, *P*_o_ was gradually decreased in a time-dependent manner and sustained at a level at 15 to 25 min upon backfilling. The inhibition showed dose-dependent decreases in channel *P*_o_ (*e.g.*, *P*_o_ was decreased from 73.6% to 47.1% by 50 nM and was further decreased to 13.8% by 100 nM GsMTx4 for this particular patch recording, Fig. 5*A*). Under the same conditions, 50 nM Pept 02 inhibited SAKcaC *P*_o_ from 72.5% to 22.1% by 50 nM and was further decreased to 6.8% by 100 nM Pept 02 (Fig. 6*B*), suggesting the dose-dependent effect for Pept 02 on SAKcaC. In fact, Pept 02 showed an even more potent inhibitory effect on SAKcaC (*e.g.*, SAKca *P*_o_ were decreased from 72.5% to 22.1% by 50 nM Pept 02 *versus* from 73.6% to 47.1% by the same concentration of GsMTx4, Fig. 5, *A*, and *B*). In addition, the dose-dependent curve for Pept 02 was leftward shifted when compared with that of GsMTx4, suggesting the decreased dissociation constant *K*_*d*_ value (required to inhibit half of the maximum channel activity) for Pept 02 (*e.g.*, the *K*_*d*_ value was 23.78 ± 6.5 nM at −50 mV for Pept 02 *versus* 47.22 ± 5.9 nM for GsMTx4 under the same conditions (Fig. 5, *C* and *D*). These results demonstrated the more potent efficacy of Pept 02 than GsMTx4 on SAKcaC.

We also studied the voltage-dependent effect of Pept 02. For this purpose, SAKcaC *P*_o_ was recorded at different voltages (from −150 mV to ∼+50 mV) before and after peptide application. Figure 5*E* shows that the same concentrations (50 nM) of both Pept 02 and GsMTx4 caused a rightward shift in the *P*_o_–*V* curves, indicating the voltage-dependent inhibitory effects for both peptides on SAKcaC. As the *P*_o_–*V* curve for SAKcaC was shifted in the midpoint voltage (*V*_1/2_) to a more positive value by 50 nM Pept 02 than that by GsMTx4 under the same conditions (Fig. 5*F*), these results suggested again the stronger efficiency for Pept 02 on SAKcaC when compared with that of GsMTx4. In addition, we noticed that the synthetic Pept 02 inhibited SAKcaC less at the membrane-depolarized conditions than that under hyperpolarized/resting states (*e.g.*, 50 nM Pept 02 inhibited SAKcaC *P*_o_ approximately ∼71.3% at −50 mV, but only ∼10.3% at + 30 mV, Fig. 5*E*), consistent with the voltage-dependent inhibitory effect observed previously for the parent peptide GsMTx4 on SAKcaC (12).

### The charged residues are essential for peptide function

The positively charged residues in peptide GsMTx4 have been suggested to be involved in direct peptide channel–lipid interaction (12, 31). We next investigated the functional roles of the positively charged residues in the shortest peptide (Pept 02) identified in this study. Structurally, Lys^2^ forms a positively charged protrusion surrounded on the surface located in the middle of Pept 02, a position directly following the hydrophobic head (formed with Trp1, the *inset* in Fig. 6*A*), thus, it is not difficult to imagine that Lys^2^ may be involved in direct peptide channel–lipid interaction, following peptide partitioning into the cell membrane. We first deleted this basic residue (Lys^2^) in Pept 02 (referred to as Pept 02-1. Fig. 6*A*) and tested its efficacy on SAKcaC. We found that under the same conditions, single-channel *P*_o_ of SAKcaC was partially reduced by extracellularly applied Pept 02-1 (5 μM) in the pipette (Fig. 6*B*), and the inhibited (%) was not further enhanced even at 30 min following Pept 02-1 applied in the pipette (Fig. 6*C*). We did not observe a significant difference for peptide inhibition rate between Pept 02 and Pept 02-1 (Fig. 6*D*); however, the inhibited (%) efficacy by Pept 02-1 was significantly weakened by ∼45.2% when compared with that by Pept 02 (*e.g.*, the inhibited [%] was 95.2 ± 3.45% by Pept 02 *versus* 53.4 ± 5.3% by Pept 02-1; Fig. 6*E*). We suggested that the positively charged Lys^2^ in Pept 02 may play important roles in peptide function.

We also investigated the role of the charged residue of Arg^11^ in Pept 03 (corresponding to Arg^18^ in GsMTx4) to confirm the conclusion that Arg11 may not be involved in the peptide (Pept 02) function. For this purpose, Arg^11^ in Pept 03 was neutralized to Ala (referred to as Pept 05, Fig. 7*A*). As shown in Figure 7*B* (*left*), although SAKcaC *P*_o_ was significantly reduced by Pept 05 (5 μM) when backfilled in the pipette, the efficacy for Pept 05 on SAKcaC was not significantly different from that by Pept 03 (Fig. 7, *C* and *D*), suggesting that substitution of Arg^11^ in Pept 03 with Ala does not affect Pept 03 function. This result is also consistent with the conclusion that Arg^18^ in loop3 of GsMTx4 (corresponding to Arg^11^ in Pept 03) does not further facilitate Pept 02 function on SAKcaC (Fig. 4). Nevertheless, when all charged residues in Pept 02 (Asp^2^/Asp^7^/Asp^8^/Lys^9^/Arg^11^) were neutralized (as we designed for Pept 02-0, Fig. 7*A*), this mutant peptide completely abolished the inhibitiory effect of Pept 02 on the SAKcaC (the *right* in Figure 7, *B*, *C* and *D*). We concluded that the charged residues in Pept 2 are also essential for peptide action on SAKcaC.

Another issue to consider was how the charged residues in Pept 02 (including Asp^2^-Asp^7^-Asp^8^-Lys^9^-Arg^11^) affect peptide effects on SAKcaC. For these purposes, we designed a series of short peptides based on the amino acids in the loop of Pept 02 (we referred to as Pept 02-1, Pept 02-2, Pept 02-3, Pept 02-4, and Pept 02-5, Fig. S5*A*). Among them, only Pept 02-1 kept the same sequence in the loop as Pept 02 (Fig. S5*A*). We found that under the same conditions, even Pept 02-2, which was designed with more positive-charged residue (Lys^3^) in the loop, did not show a substantial inhibitory effect on SAKcaC (Fig. S5*B*), SAKcaC *P*_o_ was reduced ∼28.9% by Pept 02-2 (*P*_o_ was decreased from 37.3% to 26.5% at 25 min following backfilling, Fig. S5*B*). Interestingly, the inhibitory effect by Pept 02-2 was significantly weakened even compared with Pept 02-1, which consists of less positively charged residues but keeps the same residues as Pept 02 in the loop (Fig. 6). For other peptides, including Pept 02-3, Pept 02-4, and Pept 02-5 (Fig. S5*A*), although they keep the same charged residues (Asp^7^/Asp^8^/Lys^9^) in the loop, we did not observe significant inhibitory effects on the SAKcaC (Fig. S5, *C* and *D*). We concluded that the charged residues (Asp^7^/Asp^8^/Lys^9^) alone accumulated in the loop are not sufficient for the peptide function. The stronger inhibitory effect of Pept 02-1 on SAKcaC, when compared with Pept 02-2, suggested that the synthetic peptide may require a properly folded structure in the loop (just as designed for Pept 02 with Cys-Asn-Pro-Asn-Asp-Asp-Lys in the loop) for further function. Taken together, these results support the idea that the charged residues in the peptide are essential for peptide channel–lipid interaction. In addition, the folded structure in loop2 of GsMTx4 (as designed for Pept 02) may be essential for peptide function, consistent with the conclusion that loop1 in Pept 01 (corresponding to loop 2 in GsMTx4) is essential for type I (Pept 01) function (Fig. 3).

### Hydrophobic residue in Pept 02 is critical for peptide function

**Figure 8:**
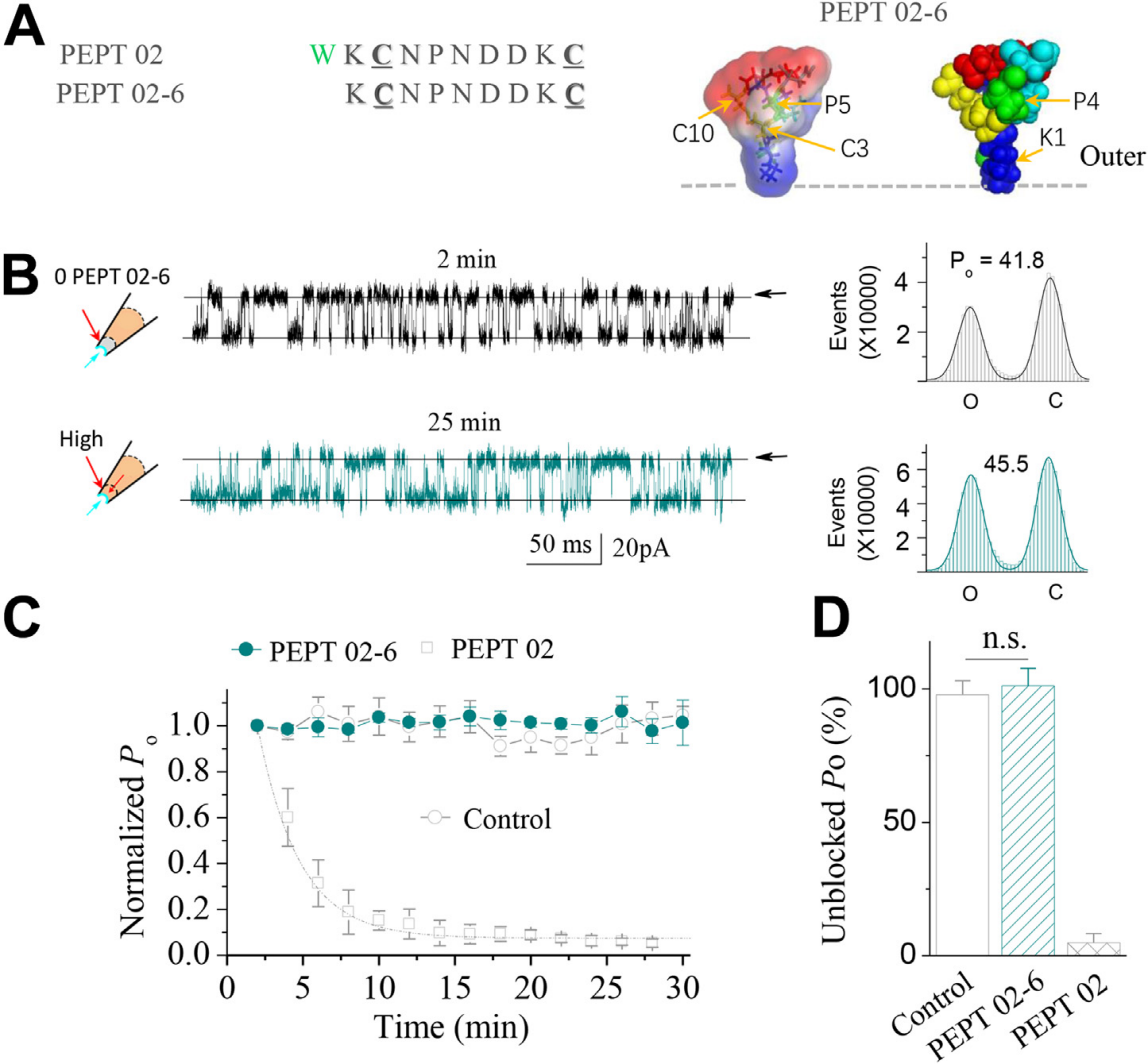
**Substitution of the hydrophobic residue in Pept 02 abolishes the inhibitory effect on the stretch-activated BK channel.***A*, sequence for the designed Pept 02-6, of which Trp-1 was substituted with Asn in Pept 02. The *inset* on the *right* shows the rendered surface image of Pept 02-6 obtained by MD simulation. *B*, the sample traces showing the inhibition effect of Pept 02-6 on the SAKcaC at the time points indicated following backfilling. The corresponding total histogram events of channel open (O) and closed (C) states were shown on the *right* with the *solid lines* fitted by the Gaussian function. Each number in the *y*-axis is timed 10,000. Time points were measured from the onset of backfilling for peptides in the extracellular side of the cell membrane. Membrane potential (*V*_m_) was held at −80 mV. *C*, time course of normalized *P*_o_ (*P*_o_/*P*_o(control)_) for Pept 02-6 during peptide diffusion to the cell membrane following backfilling at −80 mV. n = 5. The effect of Pept 02 was shown for comparison. *D*, bars represent the unblocked *P*_o_ (%) on the SAKcaC for Pept 02-6 and Pept 02. Membrane potential was held at −80 mV. Control (no peptide) was shown for comparison. Peptide concentrations used were 5 μM. ∗*p <* 0.01, ∗∗*p* < 0.005, ∗∗∗*p* < 0.001 *versus* control. ns, no significant difference *versus* control (no peptide). Intracellular calcium ([Ca^2+^]_i_) was 1 mM. SAKcaC was recorded from the CHO-expressing system. BK, big potassium; CHO, Chinese hamster ovary; MD, molecular dynamics; SAKcaC, stretch-activated big potassium channel.

The hydrophobic protrusion has been suggested to be the common feature for ICK peptide toxins, which has been presumed to facilitate peptide penetration into the cell membrane to function (27, 31). In the peptide GsMTx4, Trp^6^/Trp^7^ has been suggested to be involved in peptide partitioning into the cell membrane (31). We thus examined whether the short Pept 02 derived from loop2 of GsMTx4 is still functional on SAKcaC when the hydrophobic residue (Trp^1^, corresponding to Trp^6^ or Trp^7^ in GsMTx4) is mutated (as we designed for Pept 02-6, Fig. 8*A*). As we expected, we did not observe significant reductions in channel *P*_o_ when Pept 02-6 was applied in the extracellular side of the patch cell membrane (Fig. 8, *B* and *C*). The averaged unblocked *P*_o_ (%) by Pept 02-6 on the SAKcaC was not significantly different from the control where the peptide was not applied (Fig. 8*D*). We concluded that peptide Pept 02-6, of which the hydrophobic residue Trp^1^ was substituted with Ala in Pept 02, eliminated the inhibitory effect on the SAKcaC. Our hypothesis for the loss-of-inhibition effect for Pept 02-6 on SAKcaC is that this may arise from the lack of the hydrophobic protrusion at the head of peptide structure, such that it might lose the ability to penetrate the cell membrane for further function. The structural model for Pept 02-6 obtained with MD simulation shows that although there is still one hydrophobic residue (Pro^5^), it is not spatially located at the head (Fig. 8, the *inset* above).

**Figure 9:**
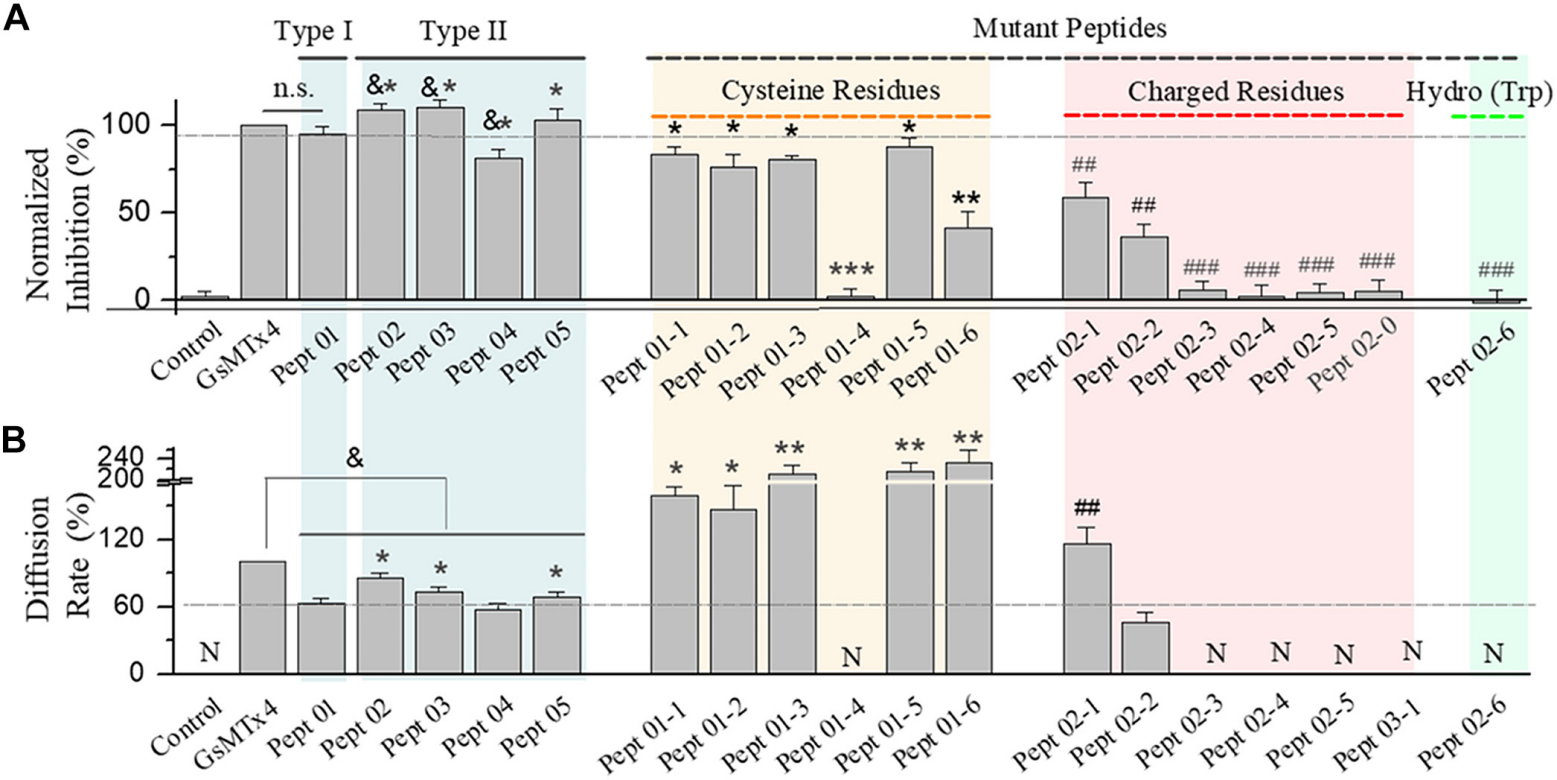
**Summary of the effects of type I, type II, and the mutant peptides on SAKcaC.***A*, comparisons of the inhibitory effects for type I, type II, and the mutant peptides on SAKcaC. The inhibitory effects were normalized to GsMTx4. Normalized inhibition (%) = [Inhibited_(peptide)_/Inhibited_(GsMTx4)_] × 100. *B*, comparisons of diffusion rates for type I, type II, and the mutant peptides on SAKcaC. The diffusion rates were normalized to GsMTx4. Diffusion rates (%) = (Diffusion rate_(peptide)_/Diffusion rate_(GsMTx4)_) × 100. (n = 4–11). &*p* < 0.05 *versus* GsMTx4, ∗*p <* 0.05, ∗∗*p* < 0.005, ∗∗∗*p* < 0.001 *versus* Pept 01. #*#p* < 0.01, ###*p* < 0.0001 *versus* Pept 02. N, no diffusion rate; ns, no significant difference. Intracellular calcium ([Ca^2+^]_i_) was 1 mM. SAKcaC was recorded from the CHO-expressing system. CHO, Chinese hamster ovary; SAKcaC, stretch-activated big potassium channel.

In summary, we have identified two types of short peptides that inhibit the SAKcaC from chick ventricular myocytes. Type I, derived from the loop2 + loop3 of GsMTx4 (*e.g.*, designed as Pept 01), showed a comparable inhibitory efficacy on SAKcaC (Fig. 9*A*) with an accelerated inhibition rate on SAKcaC when compared with the parent peptide (the inhibition rate was increased up to ∼38%, Fig. 9*B*); type II peptides, derived from the loop2 of GsMTx4 (*e.g.*, Pept 02, Pept 03, and Pept 05), showed an even more potent inhibitory efficacy on SAKcaC when compared with GsMTx4 (summarized in Fig. 9*A*). The faster inhibition rates for both type I and type II peptides (summarized in Fig. 9*B*) might arise from the smaller and sharper structures formed with Trp at the head, which has been suggested to be involved in penetrating the cell membrane for peptide GsMTx4. In addition, mutagenesis results showed that disruption of the disulfide bond (*yellow*), substitution/deletion of the charged (*pink*), or hydrophobic (*green*) residues significantly weakened or even abolished the inhibitory effects of peptides on SAKcaC (Fig. 9, *A* and *B*).

### The synthetic short peptides fail to inhibit the STREX-del mutant channel

**Figure 10:**
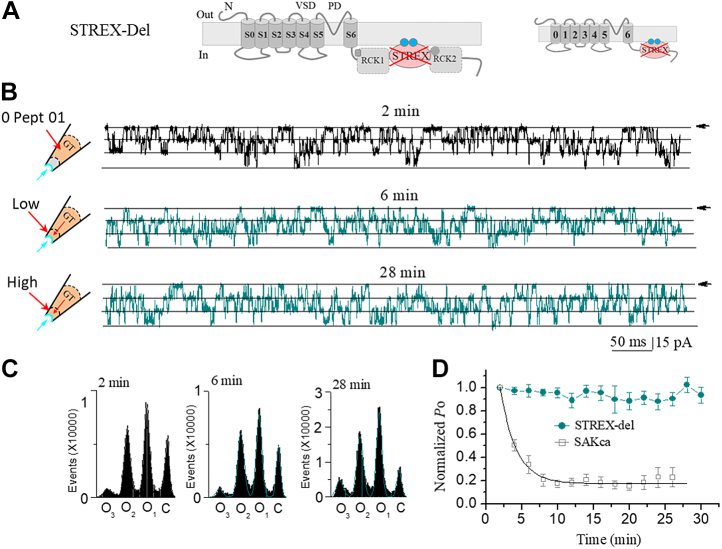
**STREX-deleted mutation (*STREX-del*) abolishes the inhibitory effect of type I (Pept 01) on the mechanosensitive SAKcaC.***A*, schematic illustrating the topology of the STREX-del mutant channel, in which the STREX domain that is attached to the plasma membrane in SAKcaC (SAKca-WT) C terminus was deleted (12, 37). *B*, sample traces showing the effect of 10 μM Pept 01 on the STREX-del channel at the time points following backfilling. The *cartoons* on the *left* represent Pept 01 backfilled in pipettes with tension (*P*_m_) automatically formed following the excised inside–out patch-clamp configuration. Time points were measured from the onset of backfilling for peptides in the extracellular side of the cell membrane. *C*, total histogram events of channels open (O_1_, O_2_, and O_3_) and closed (C) states corresponding to *B* were fitted by Gaussian functions. *D*, time courses of normalized *P*_o_ (*P*_o_/*P*_o(control)_) for during peptide Pept 01 (10 μM) diffusion to the cell membranes. *V*_m_ was held at −60 mV, n = 5. The concentration of Pept 01 used on the STREX-del mutant channel was 10 μM. The effect of Pept 01 (5 μM) on the SAKcaC was shown for comparison. SAKcaC, stretch-activated big potassium channel.

We next examined whether the two types of peptides function on the mechano-insensitive mutant channel. We have previously identified a mechano-insensitive-mutant (STREX-del) channel, of which the mechanosensor domain (STREX-exon) in SAKcaC was removed (37, 38). We also showed that GsMTx4 inhibited SAKcaC, but without effect on STREX-del channel, we thus concluded that GsMTx4 functions on SAKcaC through targeting the mechanogate (12). To investigate whether the synthetic short peptides act similarly to GsMTx4, we performed the same experiments to examine the effects of the two types of short peptides on this mutant channel. We first tested the effect of type I (*e.g.*, Pept 01) on STREX-del mutation. Although 5 μM Pept 01 caused a nearly complete inhibition on SAKcaC (Fig. 1), even a saturation concentration (10 μM) of Pept 01 did not show a significant efficacy on STREX-del mutation (Fig. 10). For example, the channel *P*_o_ of STREX-del was not significantly changed by 10 μM Pept 01 even at 28 min following backfilling in the pipette (Fig. 10, *B* and *C*). The averaged channel activities of STREX-del mutation were not significantly decreased up to 30 min (Fig. 10*D*), suggesting that this peptide inhibits SAKcaC through (directly or indirectly) targeting STREX-exon, the mechanogate for SAKcaC (12, 37). This result also indicated that the specific sites/domain for the parent peptide GsMTx4 action on SAKcaC would be located between Trp^7^ and Cys^23^ (as designed for Pept 01) in GsMTx4. We suggested that Pept 01 may mimic the parent peptide GsMTx4 to act as a mechano-sensitive BK (SAKcaC) inhibitor. Nevertheless, GsMTx4 has been suggested to act as the selective MSC inhibitor and blocks a variety of ion channels, including Na^+^ channels (45), and the MS TRPC (*e.g.*, TRPC1, TRPC6) (34) and Piezo1/Piezo2 (28) channels. Whether these short peptides act as an MS-selective inhibitor as GsMTx4 and impact the activities of these ion channels needs further investigation.

**Figure 11:**
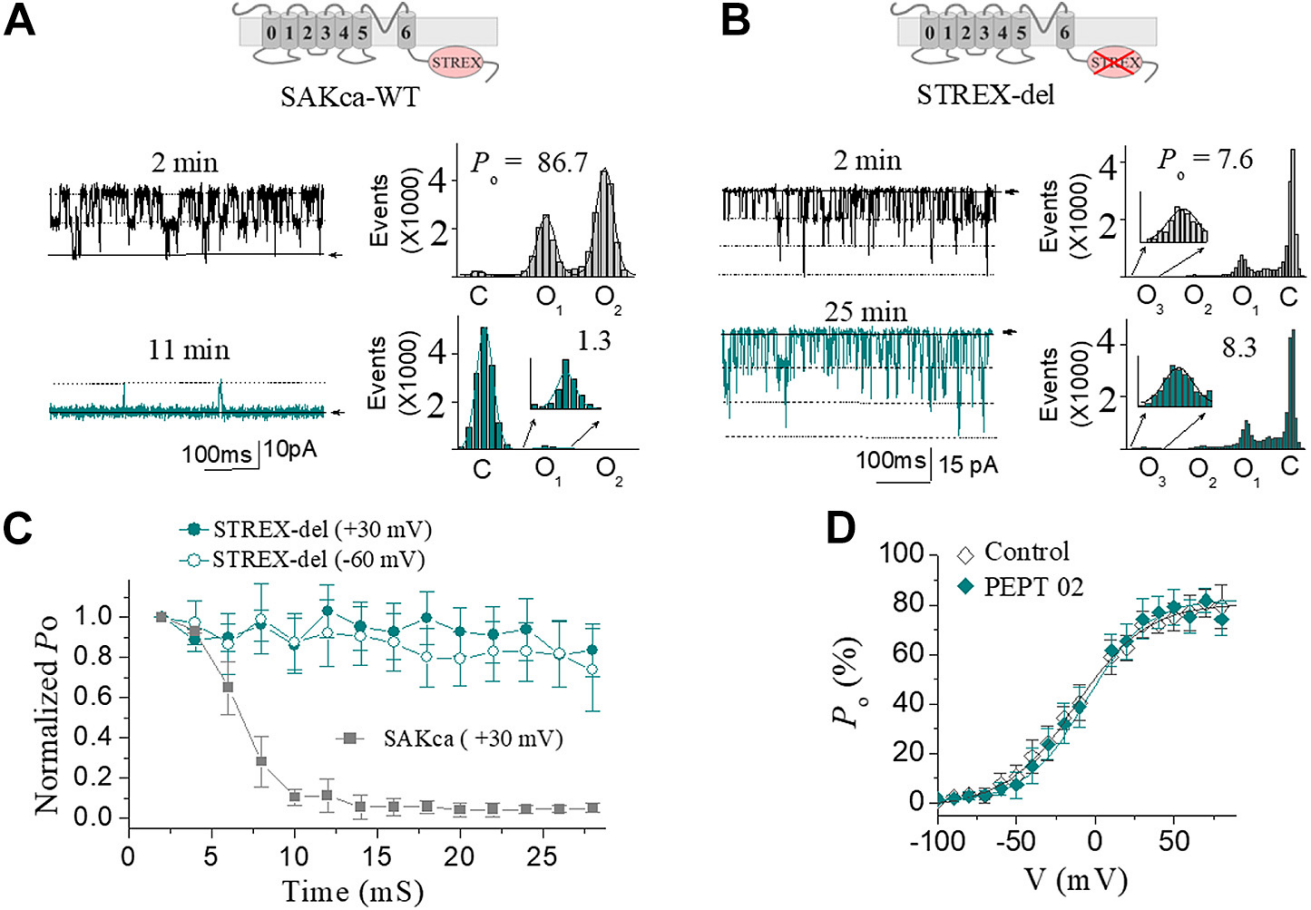
**Pept 02 failed to inhibit the STREX-del mutant channel.***A*, sample traces showing the effect of Pept 02 on the SAKca-WT channel at a depolarized voltage (*V*_m_ = +30 mV), where the peptide inhibits less on SAKcaC. The corresponding total histogram events of channel opening (O_1_ and O_2_) and closed (C) states were shown on the *right* that was fitted with the Gaussian function. The *inset* above shows the *cartoon* for the SAKca-WT. *B*, the same as in *A*, but for the effect of Pept 02 on STREX-del mutation, of which the mechanosensor domain (STREX) in SAKcaC was removed (12, 37). The *inset* above shows the *cartoon* for the STREX-del channel, where the mechanosensor domain in the C terminus of SAKcaC was deleted. MP (Membrane Potential) was held at −60 mV. *C*, time courses for the inhibition effects of Pept 02 on SAKcaC and STREX-del mutant channel at the voltages as indicated. *D*, *P*_o_–*V* relationships for the effect of Pept 02 on STREX-del channels. The *solid lines* are fit to the standard Boltzmann function. The *V*_1⁄2_ and the slope factor *K*^−1^ obtained were −9.2 ± 2.3 mV and 23.3 ± 2.8 for control (not peptide) and −5.7 ± 2.6 mV and 22.5 ± 2.1 for Pept 02. *P*_o_ was obtained 25 min following backfilling. The concentrations used for Pept 02 in *A*–*D* were 10 μM. SAKcaC were recorded from native chick ventricular myocytes, and STREX-del mutation was expressed in the CHO cell system. n = 4 to 9 per group. CHO, Chinese hamster overy; SAKcaC, stretch-activated big potassium channel.

We next tested the effect of type II peptide (Pept 02 as one example) on STREX-del mutant channel. Figure 11*A* shows that 10 μM Pept 02 dramatically decreased the *P*_o_ of SAKca-WT at the time point of 11 min following backfilling even under a depolarized membrane potential (+30 mV), where Pept 02 was shown to inhibit SAKcaC less at depolarized voltages than at hyperpolarized/resting conditions (Fig. 4*E*). Nevertheless, the channel *P*_o_ of STREX-del mutation was not significantly altered by the same concentration of Pept 02 at 25 min following backfilling in the pipette, even under a hyperpolarized/resting voltage (−60 mV) (Fig. 11*B*) where peptide was shown to inhibit SAKcaC strongly (Fig. 6*E*). The averaged channel activities of STREX-del mutation were not significantly changed by 10 μM Pept 02 as long as we recorded either at +30 mV or at –60 mV (Fig. 11*C*). The *P*_o_–*V* curve for STREX-del mutation was not significantly shifted by 10 μM Pept 02 applied in the extracellular side of the ion channel (Fig. 11*D*), indicating that even a saturation concentration of Pept 02 does not have an effect on STREX-del at all voltages ranged from −100 mV to +80 mV (Fig. 11*D*). We concluded that the mechanosensor domain (STREX-exon) in SAKcaC may act as the direct or indirect target for the short Pept 02 function. This is possible as the STREX domain located between RCK1 and RCK2 in BK C terminus has been suggested to be directly attached to the plasma membrane (39). The significantly slowed down inhibition rate by Pept 02 at +30 mV (Fig. 11*D*) *versus* under a hyperpolarized/resting state (*e.g.*, at −80 mV, Fig. 5*C*) reflects the voltage-dependent efficacy of the peptide on SAKcaC (see Discussion section). These results demonstrated that the synthetic Pept 02 (type II) inhibits SAKcaC by specifically targeting the mechanogate (STREX-exon), with stronger efficacy at hyperpolarized/resting voltage. As both types of peptides (*e.g.*, Pept 01 and Pept 02) also failed to inhibit a regular BK (mSlo1), a non-MS BK channel that lacks the STREX-exon between RCK1 and RCK2 domains (Fig. S6), we concluded that the two types of peptides (*e.g.*, Pept 01 and Pept 02), derived from toxin GsMTx4, inhibit the MS BK channel through the modulation (direct or indirect) of the mechanosensor domain (STREX).

## Discussion

This study aimed to identify short peptides that inhibit the SAKcaC through the modification specific to mechanogate. Previously, a 34-amino-acid peptide, GsMTx4, isolated from the venom of tarantula *Grammostola spatulate*, was reported to inhibit SAKcaC (12). In this study, we first identified a 17-mer short peptide (type I, referred to as Pept 01) based on the amino acids of the parent peptide, GsMTx4. This short peptide mimics the inhibitory effect of the full-length toxin without efficacy on the mechano-insensitive mutant (STREX-del) and BK (mSlo1) channels. We further identified a set of short peptides (type II, including Pept 02, Pept 03, Pept 04, and Pept 05) based on the high similarity in the backbone in the loop2 between the two mechanotoxins (GsMTx2 and GsMTx4). We showed that type II peptides showed substantial inhibitory effects on SAKcaC under the high peptide concentration (5 μM). Among them, a 10-mer short peptide (referred to as Pept 02) derived from loop2 of GsMTx4, most likely acts as an even higher-affinity inhibitor on SAKcaC when compared with the parent peptide GsMTx4. We also identified the amino acids important for the peptide function. Because the excitatory currents carried by SACs in the heart can generate fast arrhythmias (1, 6), our findings could open a way toward developing a new class of antiarrhythmic agents.

### Identification of short peptide inhibition on SAKcaC

The mechanotoxins (GsMTx2 and GsMTx4) were reported to act as the selective inhibitors for MSCs with a stronger efficacy for GsMTx4 than GsMTx2 (46). Structurally, both GsMTx4 and GsMTx2 contain six cysteines and form three pairs of cystine knots (Cys^2^-Cys^17^, Cys^9^-Cys^23^, and Cys^16^-Cys^30^), a common feature with other ICK toxins isolated from spider venom (16) (Fig. S1). Toxins that have similar physiological functions have been expected to share high homology in sequences and/or structural features (16). However, GsMTx4 shares a greater similarity in sequence to other toxins, such as VSTX1 than to GsMTx2, even though VSTX1 does not inhibit MSCs (16, 30). Similarly, the toxin ω-GsTx-SIA (ω-grammotoxin SIA), a voltage-gated Ca^2+^ Cav2.1 (P-type) and Cav_2.2_ (N-type) channel blocker, shares a higher similarity to GsMTx4 (∼33.6% identity and ∼45.8% similarity), but ω-GsTx-SIA does not have an effect on the MSCs (16, 30, 34). These correlations suggest that the linear sequence of toxins may not be sufficient for the determination of peptide functions. The higher similarity of GsMTx4 to GsMTx2 within loop2 + loop3 (16) suggested that the functional loop as well as the selectivity for toxin action on MSCs may be located between loop2 + loop3. In this study, we investigated the functional loop/segment of GsMTx4 by using an SAKcaC as a model channel. Our result that a truncated peptide, derived from loop2 + loop3 in GsMTx4 (*e.g.*, Pept 01), inhibited SAKcaC with a similar potency to the parent peptide (Fig. 1) supports this idea. The result that Pept 01 failed to function on the mechano-insensitive mutant (STREX-del) and regular BK (mSlo1) channels indicated that the functional segment/loop for GsMTx4 on SAKcaC would be located in the loop2 + loop3 of GsMTx4 (between Trp7 and Cys23).

The 3D structural analysis revealed that the backbone in loop2 between GsMTx4 and GsMTx2 is nearly superimposable (16). It is most likely that the main functional segment in loop for the GsMTx4 action may be located in loop2. Our results that under the same conditions, type II peptides derived from loop2 of GsMTx4 (*e.g.*, Pept 02, Pept 3, Pept 04, Pept 05, etc.) showed substantial and comparable efficacies on SAKcaC support this hypothesis (Figs. 4, 5, 7 and 8, also see the summary results in Fig. 9). Interestingly, among type II peptides, the shortest one (Pept 02) showed an even more potent efficacy on SAKcaC, as indicated by the increased inhibition (%) under a higher concentration (Fig. 4), the reduced *K*_*d*_ value (Fig. 5), and a stronger shift in the *P*_o_–*V* curves under a lower concentration (Fig. 5, *E* and *F*) when compared with the parent peptide GsMTx4. As the shortest peptide of Pept 02 failed to inhibit the non–mechano-sensitive mutant (STREX-del) and mSlo1 channel (Figs. 11 and S6), we suggested that loop 2 (between Trp^7^ and Lys^16^) in GsMTx4 would act as the functional selective segment/machine for GsMTx4 function on SAKCaC.

In this study, we identified two types of short peptides that inhibit SAKcaC, which contain STREX domain and confers mechanosensitivity to BK channel. In addition, we also showed that both types of peptides failed to inhibit the mechano-insensitive variant (STREX-del) as well as mSlo1. which lacks STREX domain and shows non–mechanosensitivity. It is most likely that two types of peptides act to mimic GsMTx4 on MS BK (SAKcaC) *via* targeting the STREX domain (12). Whether these two types of short peptides inhibit other MSCs (*e.g.*, the TRPC and Piezo channels (28, 34, 47, 48)) needs further investigation.

### The important amino acids required for short peptides to function

In this study, we also identified the important amino acids that are critical for the peptide function

#### Some cysteines are important for peptide action

The results that the three mutant peptides (Pept 01-1, Pept 01-2, and Pept 01-3) sustained ∼80∼90% potency of Pept 01 on SAKcaC (Fig. 1) suggested that the two cysteines (Cys^10^/Cys^11^) play minor roles for type I peptide function. Nevertheless, the further substitution of the Cys^17^ with Ala in Pept 01-3 (referred to as Pept 01-4) completely abolished the inhibitory effect of Pept 01 on SAKcaC, demonstrating that the Cys^17^ may be important for Pept 01 function in the absence of Cys^10^/Cys^11^ (Fig. 2). MD simulation results suggested that the cysteine of Cys^3^–Cys^17^ forms a disulfide bond for type I and the mutant peptides (*e.g.*, Pept 01-1, Pept 01-2, and Pept 01-3, Fig. S3), supporting the hypothesis that cystine knots in peptides act to secure the structure–function relationships (12, 27, 31). Thus, it is not difficult to imagine that disruption of the disulfide bond in Pept 01-3 (referred to as Pept 01-4) could abolish the function of Pept 01 on SAKcaC (Fig. 2).

On the other hand, the result that substitution of Cys^17^ alone in Pept 01 mostly kept the inhibitory effect of Pept 01 on SAKcaC (Fig. 3), suggested that Cys^17^ may not be required for peptide function. Nevertheless, the result that substitution of Cys^3^ alone to Ala in type I peptide mostly abolished peptide function demonstrated that Cys^3^ is essential for type I peptide function on SAKcaC.

#### The charged residues play important roles for peptide function

The positively charged residues in GsMTx4 have been suggested to be involved in peptide channel–lipid interaction (31). To investigate the functional roles of charged residues in the synthetic short peptide, we made a series of mutant peptides from Pept 02 (Figs. 6, S5, and 7). The result that deletion of Lys^2^ in Pept 02 largely decreased the inhibitory effect of Pept 02 on SAKcaC (Fig. 6) indicated that the positively charged residue of Lys^2^ is associated with peptide channel–lipid interaction. Structurally, lys^2^ in Pept 02 is located directly following the hydrophobic residue Trp^1^. Thus, it is possible that Lys^2^ may act to directly/indirectly interact/bind with channel/lipid following insertion of Trp^1^ into the lipid bilayer. The result that neutralization of all charged residues eliminated the activity of peptide on SAKcaC (Fig. 7, also see the summary results in Fig. 9) supports the idea that the charged residues in the peptides play essential roles for peptide function.

To better understand the functional roles of the charged residues in the shortest Pept 02, we designed a series of mutant peptides (Fig. S5). The result that Pept 02- (which keeps all originally charged residues of Asp^7^/Asp^8^/Lys^9^ in the loop) did not produce a significant efficacy on SAKcaC suggested that the charged residues (*e.g.*, Asp^7^/Asp^8^/Lys^9^) alone in the loop are not sufficient for peptide function. In addition, although Pept 02-4, Pept 02-3, and Pept 02-2 contain more positive charged residue (Lys following Cys) in the loop, they did not produce significant (*e.g.*, Pept 02-4 and Pept 02-3) or substantial (*e.g.*, Pept 02-2) efficacies on SAKcaC. The result that Pept 02-1, which has fewer charged residues in the loop compared with Pept 02-2, shows an even stronger inhibitory effect on SAKcaC than Pept 02-2 suggested that a suitable folded structure (*e.g.*, Asn-Pro-Asn-Asp-Asp-Lys) in the loop may be required for peptide function.

**Figure 12:**
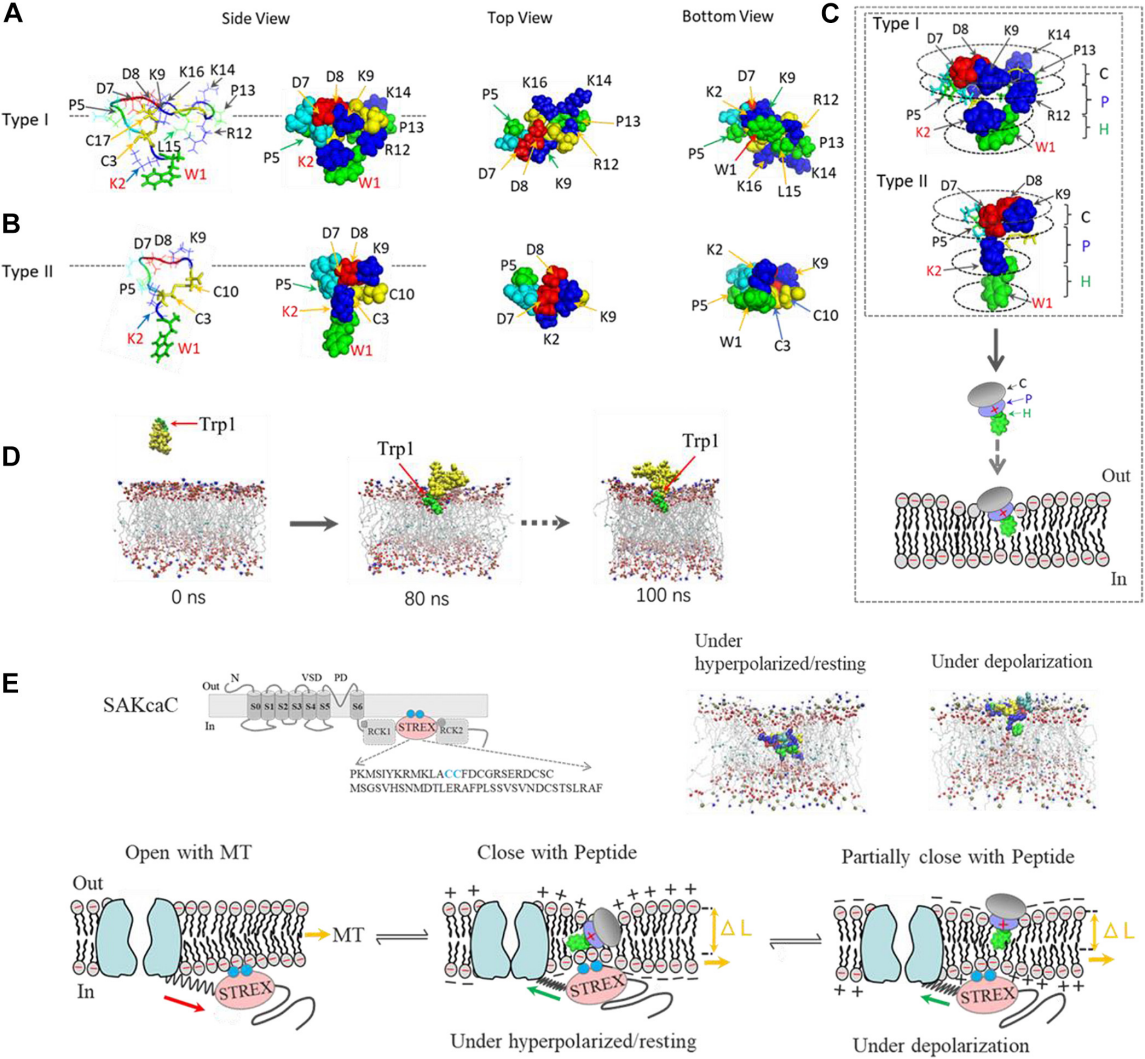
**Structural features for synthetic short peptides and the proposed gating modes for SAKcaC.***A* and *B*, the surface structures for type I (Pept 01, *A*) and type II peptides (only Pept 02 are presented, *B*). *Left* (*side views*), where Trp^1^ forms the hydrophobic protrusion at the peptide head and the positive residues (*e.g.*, Lys^2^/Arg^12^ in Pept 01 and Lys^2^ in Pept 02) form a positively charged protrusion directly following Trp1. *Middle* (*top views*); *right* (*bottom views*). The *side views* are presented based on the orientation of GsMTx4 with trypotophan (Trp) facing the intracellular side (from the outside to the inside) of the cell membrane, where Trp was assumed for GsMTx4 to be involved in peptide penetration into the cell membrane. *C*, the simplified peptide structural models showing the predominant features of both types of peptides and the supposed peptide action with the lipid bilayer (below). Note that both types of peptides contain a hydrophobic head (indicated as “H”) formed with Trp, a positively charged protrusion directly following H (indicated as “P”), and a peptide cap at the ending (indicated as “C”). *D*, MD simulation revealed that peptide has the ability to insert into the lipid bilayer with the hydrophobic head (*green*) down and induce membrane deformation. Snapshots were taken from a free simulation of type I (Pept 01) with a POPC bilayer membrane at the time points indicated. *E*, proposed gating modes modulated by membrane tension (MT) (*left*) and short peptide (*middle* and *right*) for SAKcaC: MT first pulls SAKcaC gate opening through the interaction element between STREX-lipid, where STREX domain in BK channel targets the plasma membrane by palmitoylation of two cysteine residues (*blue spheres*) (39, 40); under hyperpolarized/resting conditions (*middle*): the negative charges accumulated in the inner monolayer could attract electrostatically the positively charged protrusion in peptides (*purple*) upon its portioning into the cell membrane, thus facilitating peptide moving down (*move inward*) along the electrochemical gradients. The peptide was placed at a deep position to interact with both inner and outer monolayers, where the inner cell membrane has been suggested to interact with STREX in BK channel (*blue spheres*) (39, 40) and induce a stronger membrane deformation (also see *inset* above in the *middle*), to close the channel gate firmly; *Under depolarized state (right)*, the positively charged protrusion in peptides (*purple*) was repelled/driven back (*move outward*) by membrane potential. Alternatively, the outward electrostatic forces may also prevent the absorption of peptides from the extracellular side into the lipid bilayer. Thus, the peptide was placed at a shallow position to interact with the outer monolayer (also see the *inset* above on the *right*) and induced less membrane deformation. The schematic of the inset above (*left*) illustrates the topology of SAKcaC, where the STREX-exon located between RCK1 and RCK2 domains in the intracellular C terminus is attached to the plasma membrane by palmitoylation of the two cysteines (highlighted in *green*) (39). The snapshots in the insets above (*middle* and *right*) represent the peptide (Pept 01)–POPC bilayer interaction under the hyperpolarized/resting (*middle*) *versus* depolarized (*right*) conditions (see the Experimental procedures section) (12). Note, that the positively charged protrusions (*blue*) interacted directly with the negatively charged carbonyl oxygen atoms in the monolayer in both modes. The gating mechanism modes for SAKcaC are primarily based on the spring model proposed for BK channel (50, 58). BK, big potassium; MD, molecular dynamics; POPC, 1-palmitoyl-2-oleoyl-*sn*-glycero-3-phosphocholine; RCK, regulator of K^+^ conductance; SAKcaC, stretch-activated big potassium channel.

Interestingly, Pept 01 structure obtained with MD simulation revealed an interesting aspect that the positively charged protrusions formed with Lys^14^ and Lys^16^ (which might be involved in peptide channel–lipid interaction) are structurally located far from the hydrophobic head (formed with Trp1), suggesting that both Lys^14^ and Lys^16^ in Pept 01 may contribute little/less to Pept 01 function. Nevertheless, whether Lys^14^/Lys^16^ contribute to peptide–lipid interaction (*e.g.*, interaction with the negatively charged carbonyl oxygen atoms in the monolayer) under certain conditions (*e.g.*, Fig. 12*E*, the *inset* in the *middle*) requires further investigation.

#### The hydrophobic residue (Trp) is necessary for peptide function

Hydrophobic/aromatic residues in peptide GsMTx4 have been suggested to be involved in peptide partitioning into cell membrane for further function (29, 31). Our result that substitution of Trp^1^ with Ala in Pept 02 (referred to as Pept 02-6, Fig. 8), completely abolish peptide activity on SAKcaC, demonstrated that tryptophan (Trp^1^ in Pept 02) may be necessary for peptide function.

### Common structural features between type I and type II peptides

One puzzling question is why and how these two types of short peptides (*e.g.*, Pept 01, Pept 02, Pept 03, Pept 04, and Pept 05, etc.) function on SAKcaC similarly, as they are different in both length and sequences. To address this question, we performed MD simulations to study the structural basis and investigate the possible common features among these short peptides.

The structural models obtained by MD simulation for type I (Pept 01) and type II (Pept 02 as one example) are shown in Figure 12, *A* and *B* (others are summarized in Fig. S7). Three common features are immediately apparent from the structural models: (1) one disulfide bond formed with two cystines in each peptide is observed, which is expected to act to constrain peptide structure for ICK family peptides (16) (the disulfide-bonding patterns were Cys^3^-Cys^17^ for type I [Pept 01] and Cys^3^-Cys^10^ for type II peptides). (2) A hydrophobic head (labeled as “H”) formed with tryptophan in each peptide is particularly striking (*e.g.*, Trp^1^ for both type I and type II peptides, except Trp^0^/Trp^1^ for Pept 04, Figure 12, *A* and *B*, also see the Fig. S7), they share an identical spatial position at peptide head in each structure, a common feature with the parent peptide GsMTx4 (29), where Trp^6^/Trp^7^ in GsMTx4 (corresponding to Trp^1^ or Trp^0^/Trp^1^ in short peptides) were suggested to be involved in peptide penetrating into the cell membrane. (3) A positively charged protrusion (labeled as “P”) located directly following the hydrophobic head (formed with Lys^2^/Arg^12^ in type I of Pept 01, Lys^2^/Arg^11^ in Pept 03, and Lys^2^ in others, Fig. 12, *A* and *C*, also see Fig. S7). These positively charged protrusions seem important for peptide channel–lipid interaction. Whether the negatively charged protrusions formed with the two negatively charged Asp^8^/Asp^9^, located at the end in the peptide cap (labeled as “C,” Fig. 12*C*, also see Fig. S7), contribute to peptide function requires further investigation.

Interestingly, MD simulation revealed that the short peptide has the ability to insert into the cell membrane with the hydrophobic head down (Fig. 12*D*) and induce membrane deformation, just as observed for the parent peptide GsMTx4 (31, 49). Notably, the structural similarity in the loop1 of Pept 01-5 (folded between Cys^3^ and Cys^10^/Cys^11^, Fig. S4*B*) and that in Pept II (*e.g.*, Pept 02, folded between Cys^3^ and Cys^10^, Fig. 12*B*) supports the idea that loop2 in GsMTx4 (corresponding to loop 1 in type I peptide) play an essential role for peptide function.

Based on the aforementioned observation, we hypothesize that following the penetration of the hydrophobic head (H) of short peptides into the lipid bilayer, the positively charged protrusion (P) on the surface of each peptide structure subsequently interacts with the carbonyl oxygen atoms at the outer monolayer, which may cause substantial deformation of the lipid bilayer (the snapshot from an MD simulation in Fig, 12, *C* and *D*, also see Fig. 12*E*, the *insets* on the *middle* and *right*). It may act to decrease the energy required to deform the boundary lipids adjacent to the channel as suspected for the bilayer-dependent mechanism for GsMTx4 (33, 49).

### The proposed gating modes for SAKcaC modulated by membrane tension and the short peptides

In this study, we have presented data to show that both types of synthetic peptides (*e.g.*, Pept 01 and Pept 02) inhibit SAKcaC through specifically targeting STREX-exon (the mechanogate) as in the case of the effect of the full-length peptide GsMTx4 on SAKcaC (12). We also studied the detailed properties for the inhibitory efficacy of the shortest one (Pept 02) on SAKcaC. Our hypothesis for the faster inhibition rate for Pept 02 under hyperpolarized/resting states *versus* under depolarized conditions (Figs. 4*C* and 11*C*) may arise from different electrostatic interaction modes.

Previously, we have shown two binding modes for GsMTx4 channel–lipid bilayer interaction followed by a free 200-ns production run of MD simulation (12); under hyperpolarized/resting conditions, the positively charged GsMTx4 was driven down along the electrochemical gradients (move inward) upon partitioning into the lipid bilayer; it was placed at a deep position to interact with both inner and outer monolayers and induce strong membrane deformation, resulting in channel gate closed firmly; whereas under the depolarized state, GsMTx4 was driven back (move outward) by membrane potential, it was placed at a shallow position to interact with the outer monolayer only, thus resulting in a less/weak membrane deformation. These two gating modes were suspected based on the idea that the net charge of GsMTx4 is positive (+5). Nevertheless, Pept 02 is neutral, it functions on SAKcaC similarly to both Pept 01 and GsMTx4, where the net charges for both are positive (the net charge for Pept 01 is +3). Based on the common features observed in the structures between type I (*e.g.*, Pept 01) and type II (*e.g.*, 02) peptides (Fig. 2, *A*–*C*), we proposed two modes for their actions on SAKcaC: membrane tension first pulls SAKcaC gate opening *via* STREX–membrane interaction (Fig. 12*E*, *inset* on the *left*) (39) to turn the passive spring of BK gating ring (RCK1–STREX–RCK2 in SAKcaC) into a force-generating machine and opens channel gates (Fig. 12*E*, *left*) (12, 50).

Under hyperpolarized/resting states, where positive charges are accumulated in the outer monolayer (the extracellular side of the cell membrane) and the negative charges in the inner monolayer (the intracellular side of the cell membrane), the positively charged protrusion (P), located directly following the hydrophobic head, is absorbed down along the electrochemical gradients (from the outer to the inner lipid bilayer) upon the hydrophobic head (H) partitioning into the lipid bilayer. Alternatively, the inward electrostatic forces may also promote the absorption of the positively charged protrusion in the peptide from the extracellular side into the lipid bilayer, such that the peptide is subsequently placed at a deep position to bind simultaneously with both outer and inner leaflets of the lipid bilayer and induces strong membrane deformation. This generates a strong closing force to push STREX back strongly *via* the interaction between STREX and plasma membrane (39) to close channel gate firmly (Fig. 12*E*, *middle*). This mode is similar to the deep binding mode proposed previously for GsMTx4 action (12). However, in this mode, the short peptide is assumed to be stabilized deeply by the interaction of the positively charged protrusion (formed with Lys^2^/Rrg^12^ in Pept 01and Lys^2^ in others) tightly with the negatively charged carbonyl oxygen atoms in both leaflets of the lipid bilayer as shown in Fig. 12*E* (*middle*, also see the *inset* in the *middle*).

Under membrane depolarization conditions (Fig. 12*E*, *right*), the positively charged protrusion (formed with Lys^2^/Rrg^12^ in Pept 01 and Lys^2^ in others) is repelled/driven back (from Fig. 12*E*, *middle* to *right*) along the electrochemical gradients(move outward); the closing forces generated by peptide under the hyperpolarized/resting conditions (Fig. 2*E*, *middle*) are certainly released, thus resulting in less inhibitory effect for peptide on SAKcaC, unless a high concentration is used for the positive protrusion to bind/neutralize the negative charges accumulated outside the outer monolayer (the extracellular side of the cell membrane). The result that a higher concentration of Pept 02 (10 μM) completely inhibited SAKcaC at the depolarized condition supports this idea (Fig. 11, *A* and *C*). In addition, the outward electrostatic forces generated by membrane depolarization may also prevent the absorption of the positively charged protrusion from moving down against the electrochemical gradients, resulting in less closing force on the inner leaflet of the cell membrane. This mode is corresponding to the shallow binding mode proposed for GsMTx4 action (12). In these proposed gating modes, an interaction element of STREX-lipid bilayer was suspected to act as the communicator for peptide action on SAKcaC (39). Specifically, since the STREX domain in BK C terminus has been suggested to target the plasma membrane by palmitoylation of the cysteines (Fig. 12*E*, *inset* on the *left*) (39), we thus hypothesized that the interaction element (STREX–lipid bilayer) would act as the communication for peptide inhibition on SAKcaC. It is possible that following the partitioning into the cell membrane, the peptides may selectively target the STREX domain in SAKcaC *via* the STREX–lipid element, where following the partitioning into the cell membrane, the hydrophobic head interacts directly with the lipid bilayer. The positively charged residues (*e.g.*, Lys^2^) in the peptide may also interact with the negatively charged carbonyl oxygen atoms in the leaflets of the lipid bilayer to promote the interaction between the peptide–lipid bilayer and target with STREX domain in SAKcaC C terminus for function.

Based on the two modes proposed for SAKcaC gating, it is not difficult for one to imagine that deleting/neutralizing the positively charged residues in the peptide could disrupt the interaction component of peptide–lipid–STREX, thus disrupting/reducing peptide efficacy. The results that deletion of Lys^2^ or substitution of the charged residues in Pept 02 decreases/eliminates the inhibitory effect (Fig. 6) support this idea. Alternatively, as the net charges for both Pept 01 and Pept 03 are positive, a similar mechanism proposed for GsMTx4 on SAKcaC may also apply to both short peptides.

In summary, in this study, we identified two types of short peptides that show similar (*e.g.*, Pept 01) or even more potent inhibitory effects (*e.g.*, Pept 02, Pept 03, and Pept 05) on SAKcaC from chick hearts, when compared with the parent peptide GsMTx4. As both types of short peptides failed to inhibit STREX-Del mutant as well as the regular BK (mSlo1), a STREX-lacking/non-MS BK channel, we suggested that both types of short peptides mimic GsMTx4 to inhibit the SAKcaC through the specific modulation to the mechanogate (STREX-exon). It has been shown that the spider venom GsMTx4 inhibits the stretch-induced AF to help hearts keep their rhythm (1, 7), but whether the short peptides identified in this study mimic GsMTx4 to inhibit AF needs further identification. Our finding may provide a new way to develop a new type of antiarrhythmic inhibitor that mimics GsMTx4 to prevent the heartbeat from losing its rhythm, as GsMTx4 does (1, 7).

## Experimental procedures

### Mutagenesis and channel expression

The SA BK (SAKca) channel (SAKcaC) gene was cloned from the complementary DNA (cDNA) library (GenBank accession number: AB072618) from chick embryonic hearts (13); this was further subcloned into mammalian expression vectors (pcDNA 3.1). SAKca STREX (stress axis–regulated exon) deletion mutation (STREX-Del) was verified by sequencing as published previously (35, 51). Cloned SAKcaC (SAKca-WT) or STREX-Del mutant cDNAs were transiently transfected in the CHO-K1 cells using the Lipofectamine 2000 Transfection Reagent (Thermo Fisher Scientific) according to the manufacturer’s protocol. To monitor the successfully transfected cells, pEGFP (Clontech Laboratories) was coexpressed with the SAKca-WT or STREX-Del channel cDNA (5:1, w/w). The transfected cells grown on the coverslip were used for patch-clamp recordings. No endogenous BK channel activity was detected in the untransfected CHO-K1 cells. Currents were normally recorded within 1 week after cDNA transfection (13, 37, 38).

Native SAKcaCs were recorded from ventricular myocytes, which were isolated from chick hearts as described previously (12, 13, 37).

### Electrophysiology

Single-channel currents for SAKcaC were recorded under the standard excised inside–out patch configuration. Currents were amplified using an A&M amplifier (Model 2400) or an EPC10 patch-clamp amplifier (HEKA Elektronik). Data were filtered at 10 kHz. The standard pipette solution facing the extracellular surface of the patch contained (in millimolar): 145 potassium gluconate, 1 EGTA, 10 Hepes, and 5 glucose, pH 7.4 was adjusted with NaOH. The bath solution facing the cytoplasmic surface of the patch membrane was the same except the [Ca^2+^]_i_ concentration used was 1 mM (13, 38).

Macroscopic currents for regular BK (mSlo1) channels were recorded from *Xenopus laevis* oocytes by using standard excised inside–out patches. mSlo1 cDNA was a gift from the laboratory of Dr Christopher Lingle (Washington University), of which the vector pXMX was designed to promote circular RNA expression (12, 36, 52). pClamp (Molecular Devices) was used to drive stimulus protocols and digitize currents as described previously (51, 53). The standard pipette (extracellular) solution contains (in millimolar): 140 KMES (methanesulfonate), 20 KOH, 10 Hepes, 2 MgCl_2_, and pH 7.0. The internal solution contains (in millimolar) 140 KMES, 20 KOH, 10 Hepes, 5 EGTA, and pH 7.0. The free intracellular [Ca^2+^]_i_ solution was buffered as described previously (36, 52).

As the parent peptide, GsMTx4 did not show a significant effect on the MS BK channel when applied in the bath (the intracellular side of ion channel) (12), all peptide effects were applied from the extracellular side of the cell membrane using the standard backfill method established previously (12, 42, 43). In brief, the normal pipette solution was first filled in the tips of the electrodes, and then the same solution containing the peptide was backfilled. Since we did not observe significant effects on SAKcaC at a lower concentration (100 nM) for some mutant peptides (*e.g.*, Pept 02-1, Pept 02-2), all peptide effects were compared at the concentration of 5 μM, except otherwise noticed. All experiments were performed with standard inside–out patch configuration at room temperature (22–25 °C).

### Single-channel analysis

Single-channel conductances of SAKcaC and the STREX-del mutant channel were determined by the slope of current–voltage (I–V) curves, where I–V data could be well fitted with a linear function (12, 54). Amplitude histograms were measured in inside–out patches mostly with one to three channels. Histograms were fitted with a Gaussian function using pClampfit software (Molecular Devices Corp.). In the case when only one channel was contained in patches, channel open probability *P*_o_ (%) was determined simply by driving the total open duration of the sum of the open and closed times in patches as described previously (51, 55). When multiple channels were presented in the patch, *P*_o_ was determined using the area under each peak (*aj*) at each current level (*j*) in the histogram along with the number of channels (*N*):

(1) $$Po=\frac{\sum_{j=0}^{n}\left(j*aj\right)}{n\times \sum_{j=0}^{N}aj}$$

Where *N* is the channel number in each patch, which was defined from the maximum level observed at a relatively higher voltage of +80 mV under 1 mM [Ca^2+^]_i_.

### Structural models for synthetic short peptides

The 3D model structure for short peptides was established by using visual molecular dynamics simulation (12, 54, 56). Specifically, the structural model for type I short peptides (*e.g.*, Pept 01 and its mutant peptides) were predicted based on the known structure of the neuropeptide GsMTx4 (Protein Data Bank ID: 1TYK); the structural models for type II short peptides (*e.g.*, Pept 02, Pept 03, Pept 04, and Pept 05, etc.) were developed based on Pept 01. The simulation programs, conditions, and water models were basically the same as described previously used for the mutant peptides (31). The initial box size (Å) was 65 × 65 × 80. MD simulations were performed with an ∼80 ns production run followed by an energy minimization and an equilibration run. The temperature was set at 323.15 K with Nose–Hoover coupling. The pressure was controlled by the Parrinello–Rahman at 1 atm with the independent (semi-isotropic) coupling in the *xy* and *z* directions. The MD simulation outcome for each short peptide forms one disulfide bond (disulfide-bonding pattern: Cys^3^-Cys^17^ for Pept 01 and Cys^1^-Cys^10^ for others). The systems for the simulations of lipid–peptide interaction under the hyperpolarized/resting *versus* depolarized conditions were settled in the electric fields along the *z*-axis as used as described previously (12, 57). The simulation was carried out at the National Supercomputer Center in LvLiang of China. All molecular images were made with PyMOL 2.5 (PyMOL|pymol.org).

### Peptides and chemicals

The peptide GsMTx4 was purchased from Alomone Labs. All short peptides were commercially synthesized with a purity >98% (Sangon Biotech). The aqueous stock solutions were prepared at 5 mM or 10 mM in distilled water. Pept 02-0 was dissolved in *N*,*N*-dimethylformamide with a concentration of 50 to 100 μg/μl (∼23–46 mM) and further diluted to 10 mM in distilled water for the stock solution. The stock solutions (5 mM or 10 mM) were stored at −80 °C, and an appropriate amount of the stock aliquot was diluted in normal pipette solution freshly to the concentrations used on the day when patch-clamp experiments were performed. Other chemicals were purchased from Sigma–Aldrich unless otherwise noted.

### Data analysis

Data acquisition and analysis were carried out using pClamp9 (Molecular Devices), ANA. (Molecular Devices, Corp.), and Origin 7.5 software (OriginLab). All data are presented as mean ± SEM in all figures. Statistical significance was evaluated by a Student’s *t* test. *p* ≤ 0.05 was considered statistically significant.

## Data availability

All data are contained within the article and supporting information.

## Supporting information

This article contains supporting information (Figures S1–S7).

## Conflict of interest

The authors declare that they have no conflicts of interest with the contents of this article.
