# Supplementary material for: Two types of peptides derived from the neurotoxin GsMTx4 inhibit a mechanosensitive potassium channel by modifying the mechanogate
Source: J Biol Chem. 2022 Aug 4;298(9):102326. doi: 10.1016/j.jbc.2022.102326 (PMC9449670; doi:10.1016/j.jbc.2022.102326)
Supplement: Supporting information [file mmc1.pdf]

Two types of peptides, derived from neurotoxin GsMTx4, inhibit a mechanosensitive BK channel through the modification specific to mechano-gate

Nan Zhou[1#], Hui Li[1#], Jie Xu[1#], Zhong-Shan Shen[1#], Mingxi Tang[2#], Xiao-Hui Wang[1], Wan-Xin Su[1], Masahiro Sokabe[4,5], Zhe Zhang[1,3,6*], Qiong-Yao Tang[1,3,6*],

[1]Jiangsu Province Key Laboratory of Anesthesiology, Xuzhou Medical University, Xuzhou, Jiangsu Province, China, [2]Department of Pathology, the Affiliated Hospital of Southwest Medical University, Taiping Road 25, Luzhou, Sichuan, China, [3]Jiangsu Province Key Laboratory of Anesthesia and Analgesia Application Technology, Xuzhou Medical University, Xuzhou, Jiangsu Province, China, [4] Mechanobiology Laboratory, Nagoya University. Graduate School of Medicine, Nagoya, Japan, [5]Kanazawa Institute of Technology, Nonoichi, Japan, [6]NMPA Key Laboratory for Research and Evaluation of Narcotic and Psychotropic Drugs, Xuzhou, Jiangsu Province, China.

Contents:

	Page
Figure S1. Comparison of the two mechanotoxin (GsMTx4 with GsMTx2) with homologous ICK motif toxins that are not effective on MS channels	S2
Figure S2. Pept 01 inhibits mechanosensitive BK channel (SAKcaC) without changing single-channel conductance	S3
Figure S3. MD simulation revealed that Cys^{3}-Cys^{17} pattern forms a disulfide bond regardless of whether one or two of the cysteines (Cys^{10}/Cys^{11}) were substituted	S4
Figure S4. MD simulation indicated that Cys^{3} forms a disulfide with Cys^{10}/Cys^{11} for type I (Pept 01) peptide in the case when Cys^{17} is absent	S5
Figure S5. The charged residues alone in Pept 02 are not sufficient to produce the inhibitory effect on the stretch-activated BK channel	S6
Figure S6. Both tye I (Pept 01) and type II (Pept 02) failed to inhibit the regular BK(mSlo1) channel.	S7
Figure S7. The structural comparisons for the synthetic peptides	S8

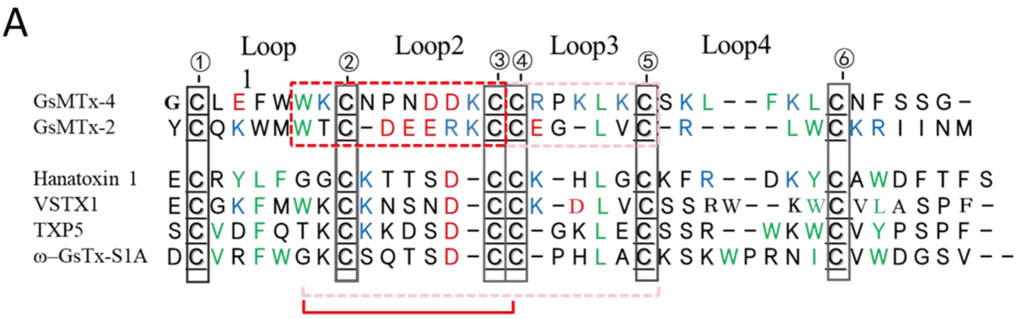

B

Toxins	Target of Ion Channel	Identity	Similarity
GsMTx2	MS	11%	28%
Hanatoxin 1	K$^+$	28%	37%
VSTX1	Voltage-dependent	43%	54%
TXP5	K$^+$	40%	49%
ω–GsTx-S1A	N-,P- and Q-type Ca^{2+}	34%	46%

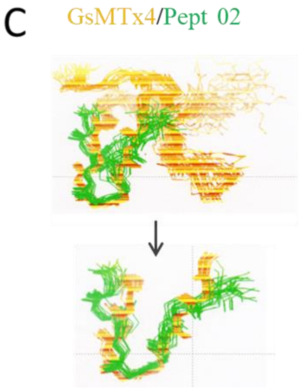

Figure S1. Comparison of the two mechanotoxin (GsMTx4 with GsMTx2) with homologous ICK motif toxins that are not effective on MS channels. (A) Sequence comparison of mechanotoxin GsMTx4 with GsMTx2 and with other homologous ICK peptides. Green highlights the conserved hydrophobic residues that combine to form a hydrophobic cluster on the folded peptides. Blue residues show the positively charged residues and red for the negative ones. (B) Comparison in sequence identity and similarity of GsMTx4 with GsMTx2 and with homologous ICK toxins ineffective on MS channels. Note the peptides, which are not effective on MS ion channels (e.g., hanatoxin, VSTX1, etc.,), share even higher sequence identity and similarity to GsMTx4 than GsMTx2. (C) The structure of the synthetic short peptide Pept 02 (yellow) is mostly superimposed with the loop2 in GsMTx4.

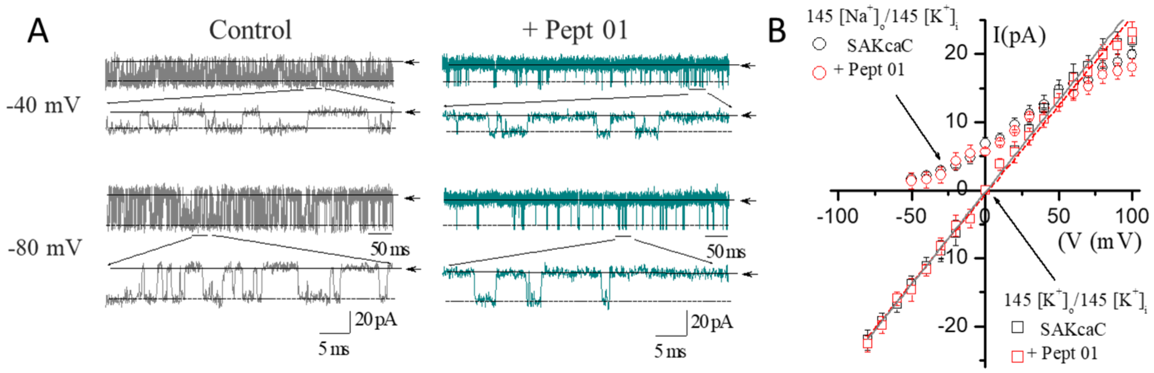

Figure S2. Pept 01 inhibits the mechanosensitive BK channel (SAKcaC) without changing single-channel conductance. (A) Typical single-channel current comparisons for SAKcaC between control (left) and in the presence of Pept 01 when backfilled in the pipette (see Method). Current traces in the presence of Pept 01 were obtained 25 min following backfilling. The intracellular $[Ca^{2+}]_i$ (in the bath) used was 1 mM(see Methods). (B) *I-V* relationships comparisons for SAKcaC in the absence (control: open symbols) and presence (filled symbols) of 5 uM Pept 01 in the pipette (see methods) with symmetrical 145K^+ (squares) or 145 $[Na^+]_o$ /145 $[K^+]_i$ (circles). Currents were recorded with 1mM $[Ca^{2+}]_I$, and SAKcaCs were recorded from the CHO-expressing system. Data points represent 4-6 experimental determinations.

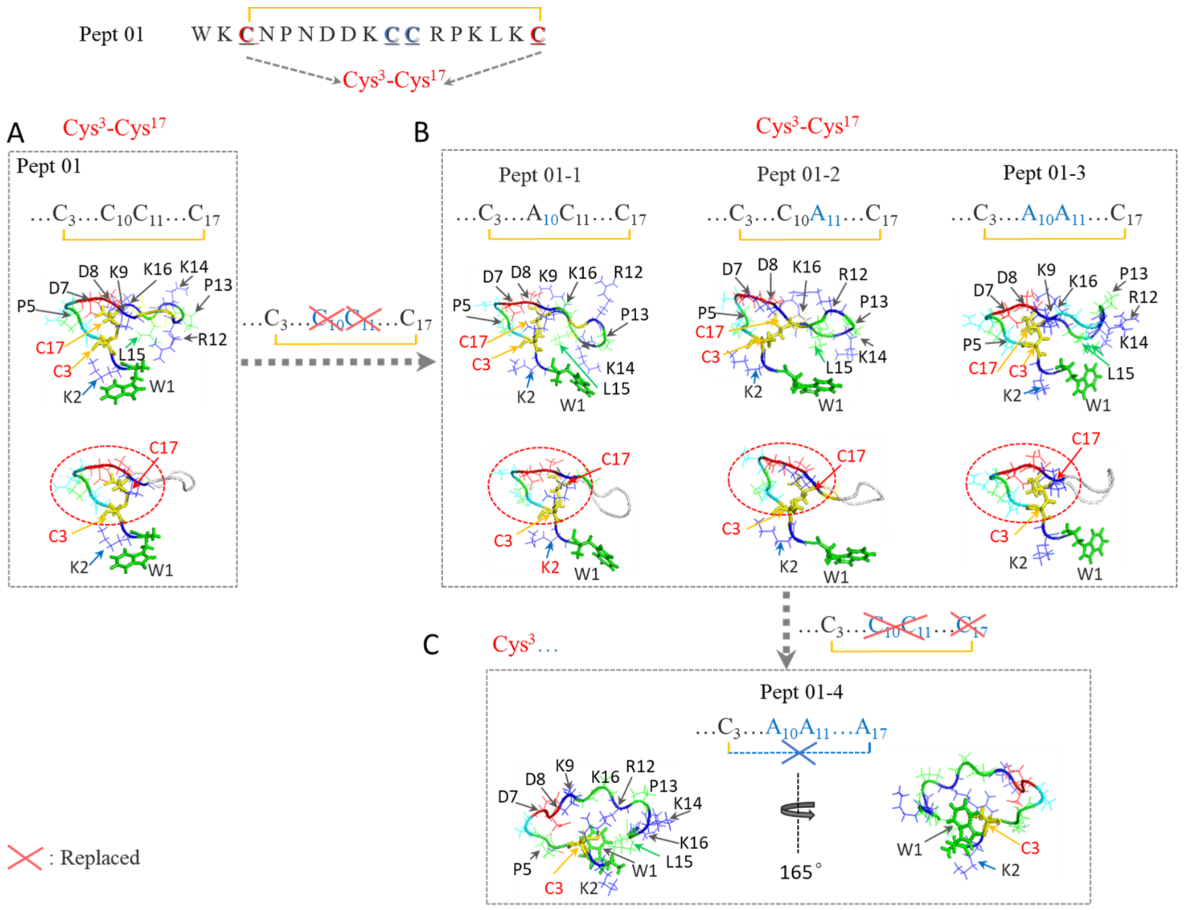

Figure S3. MD simulation revealed that Cys[3] forms a disulfide bond with Cys[17] regardless of whether one or two of the cysteines (Cys[10]/Cys[11]) were substituted. (A) The backbone structural model for Pept 01. The structural model for Pept 01 was established based on the parent peptide GsMTx4 (PDB code 1TYK) by using the MD simulation method. The aromatic and hydrophobic residues are shown in green, positively charged residues are shown in blue, negatively charged residues in red, and cysteines in yellow. The disulfide bond formed with cysteine pattern Cy3-Cys[17] is presented in sticks, which may act to constrain the peptide structure for ICK family peptides (1). Bellow: the same structural model as above, with the highlight of the structural loop fold with cysteine patter of Cys[3]-Cys[17]. (B) the same as in A, but for the mutant peptides with one (Pept 01-1, left, and Pept 01-2, middle) or two (Pept 01-3, right) cysteines (Cys[10]/Cys[11]) are substituted with Ala, which is highlighted in blue. (D) The backbone structural model for Pept 01-4, of which three cysteines of Cys[10]/Cys[11]/Cys[17] were substituted to Ala. Structures are presented with the hydrophobic head formed with Trp[1] down facing the intracellular side (from the outside to the inside) of the cell membrane. As Trp1 is corresponding to Trp[6]/Trp[7] in GsMTx4 that were suggested to be involved in peptide penetrating into the cell membrane(2). The lower panels highlight the loop folded with disulfide bond Cys[3]-Cys[17], which shares between Pept 01 and three mutant peptides (Pept 01-1, Pept 01-2, and Pept 01-3).

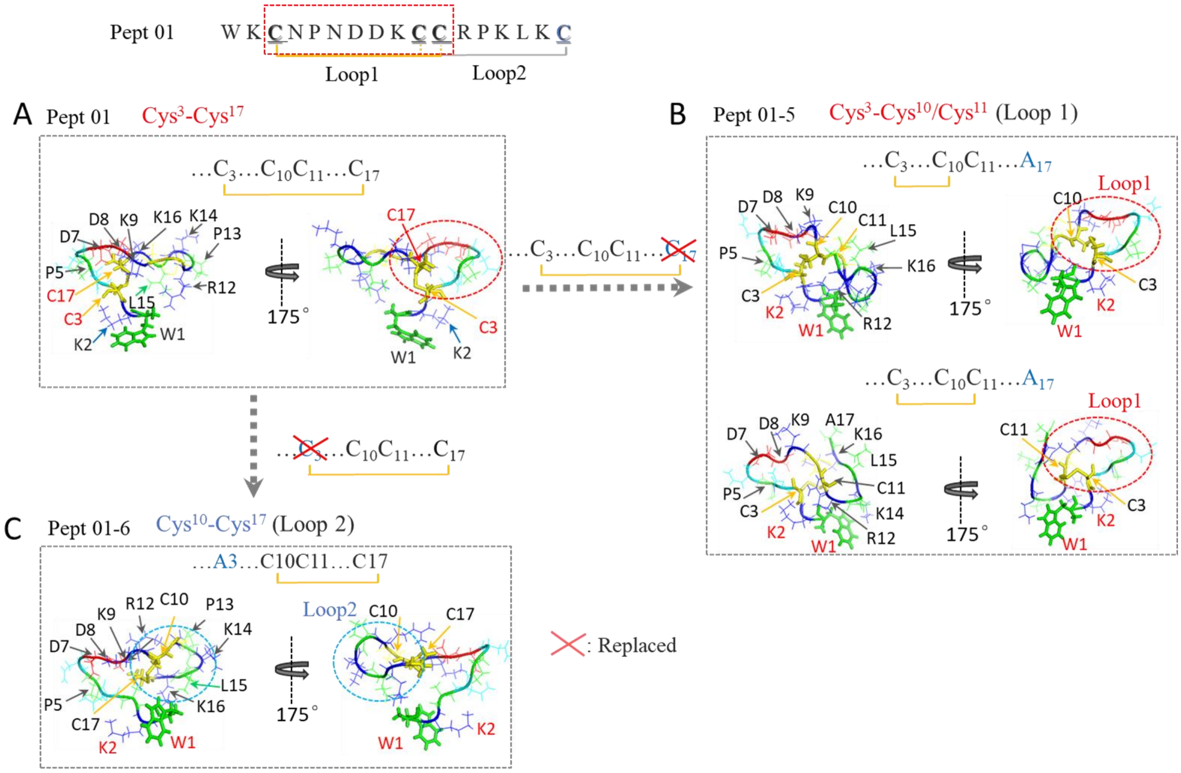

Figure S4. MD simulation indicated that Cys[3] forms a disulfide with Cys[10]/Cys[11] for type I (Pept 01) function in the case when Cys[17] is absent.

(A) The backbone structural models for Pept 01 in two different orientations as indicated. The structural models were established based on the parent peptide GsMTx4 (PDB code 1TYK) by using the MD simulation method. The aromatic and hydrophobic residues are shown in green, positively charged residues are shown in blue, negatively charged residues in red, and cysteines in yellow. The disulfide bond formed with cystine pattern Cys[3]-Cys[17] is highlighted in sticks, which may act to constrain peptide structure for ICK family peptides. (B) The same as in A, but showing two different states of the structures for Pept 01-5, of which Cys[17] is substituted with Ala (highlighted in blue). Note, the disulfide bond in each state (Cys[3]-Cys[10] for the upper one, and Cys[3]-Cys[11] for the bellow) forms a functional loop (loop1). (D) The same as in B, but for the structural model of Pept 01-6, of which Cys[3] is substituted with Ala (highlighted in blue). Note, the cystine pattern of Cys[3]-Cys[10] forms a disulfide bond which results in a functional loop (loop2) between Cys[10]-Cys[17]. The inset above shows the liner sequence of Pept 01, the cysteine knot is highlighted.

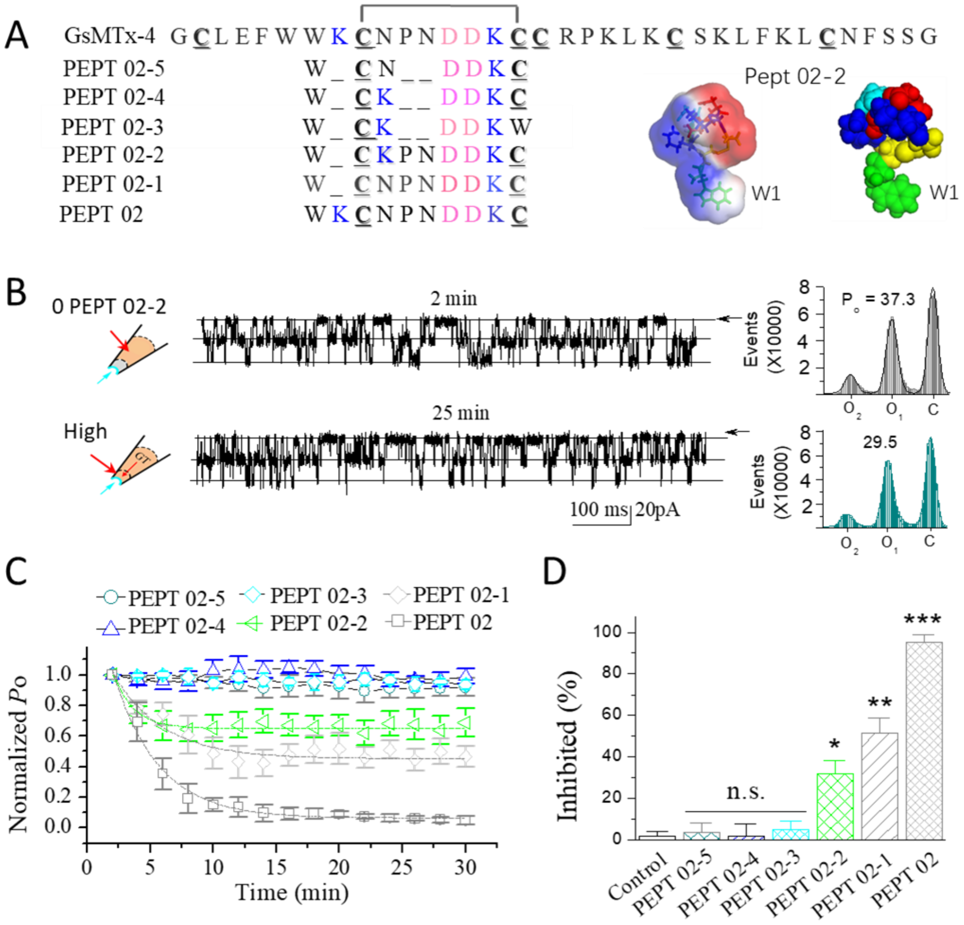

Figure S5. The charged residues alone in Pept 02 are not sufficient to produce the inhibitory effect on the stretch-activated BK channel. **(A)** Sequences for the designed peptide of Pept 02-1, Pept 02-2, Pept 02-3, Pept 02-4 and Pept 02-5. (B) The sample traces showing the inhibition effect of Pept 02-2 on the SAKcaC at the time points indicated following backfilling. The corresponding total histogram events of channel open (O_1 and O_2) and closed (C) states shown on the right were fitted to Gaussian functions. Each number in the y axis is timed 10000. The cartoons on the left represent the peptides backfilled in pipettes with tension (P_m) automatically formed following the excised inside-out patch-clamp configuration. Time points were measured from the onset of backfilling for peptides in the extracellular side of the cell membrane. Membrane potential (V_m) was held at -80 mV. (C) Time courses of normalized P_o ($P_o/P_{o\ (control)}$) for Pept 02-1, Pept 02-2, Pept 02-3, Pept 02-4 and Pept 02-5 during peptides diffusion to the cell membranes following backfilling at -80 mV. The effect of Pept 02 was shown for comparison. P_o was normalized to the level following the excised inside-out patch configuration ($P_{o\ (control):}$ before peptide diffusion to the cell membrane). (E) Comparison of the inhibited effects (Inhibited (%)) among the peptides of Pept 02-1, Pept 02-2, Pept 02-3, Pept 02-4 and Pept 02-5, Pept 02 effect was shown for comparison. Peptide concentrations used were 5 μM. *: P < 0.05; ***: P < 0.001; n.s. no significant difference *vs*. Control (no peptide backfilled). Data points represent the mean ± S.E. (error bars) of at least four experiments. Intracellular calcium ($[Ca^{2+}]_i$) was 1 mM. SAKcaC was recorded from the CHO-expressing system.

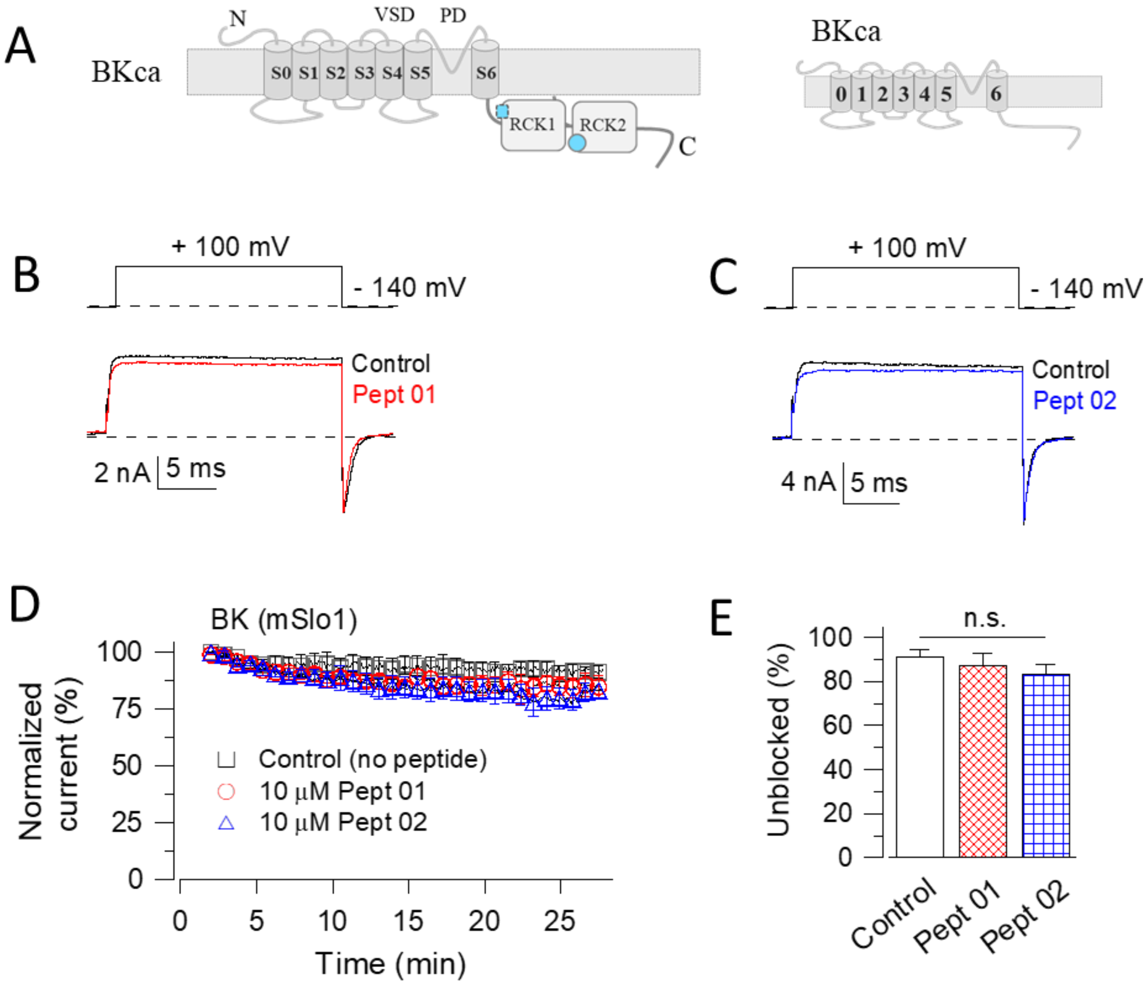

Figure. S6. Both tye I (Pept 01) and type II (Pept 02) failed to inhibit the regular BK(mSlo1) channel. (A) schematic of the regular BKca (mSlo1) channel. The transmembrane domain (S0–S6) contains a VSD (S1–S4) and a PD (P5–P6), and the cytoplasmic domain contains two RCK (*RCK1* and *RCK2*) domains, as in the case for SAKcaC, but it lacks the STREX (*pink*) in BK-terminus, the mechano-sensory domain located between the RCK1 and RCK2 domains in SAKcaC. (B) Macroscopic currents recorded for BK (mSlo1) channels in the absence (*black*) or presence (red) of Pept 01 back-filled in the pipette. (C) the same as in B, but for the effect of Pept 02 (blue) back-filled in the pipette. (D) Time courses for normalized mSlo1 macroscopic currents during Pept 01 or Pept 02 diffusion to the patch cell membranes. The time points were measured from the onset of back-filling from the extracellular side of the cell membrane. (E) Bars represent the uninhibited effects (%) for Pept 01 and Pept 02 on mSlo1, compared with control (with no peptide applied). The uninhibited (%) was obtained 25 min later following backfilling. Peptide concentrations used were 10 μM. $[Ca^{2+}]_i$ in both solutions were 100 µM. n.s. not significantly different. BKca (mSlo1) was recorded from Xenopus oocytes. n = 4-6 per group.

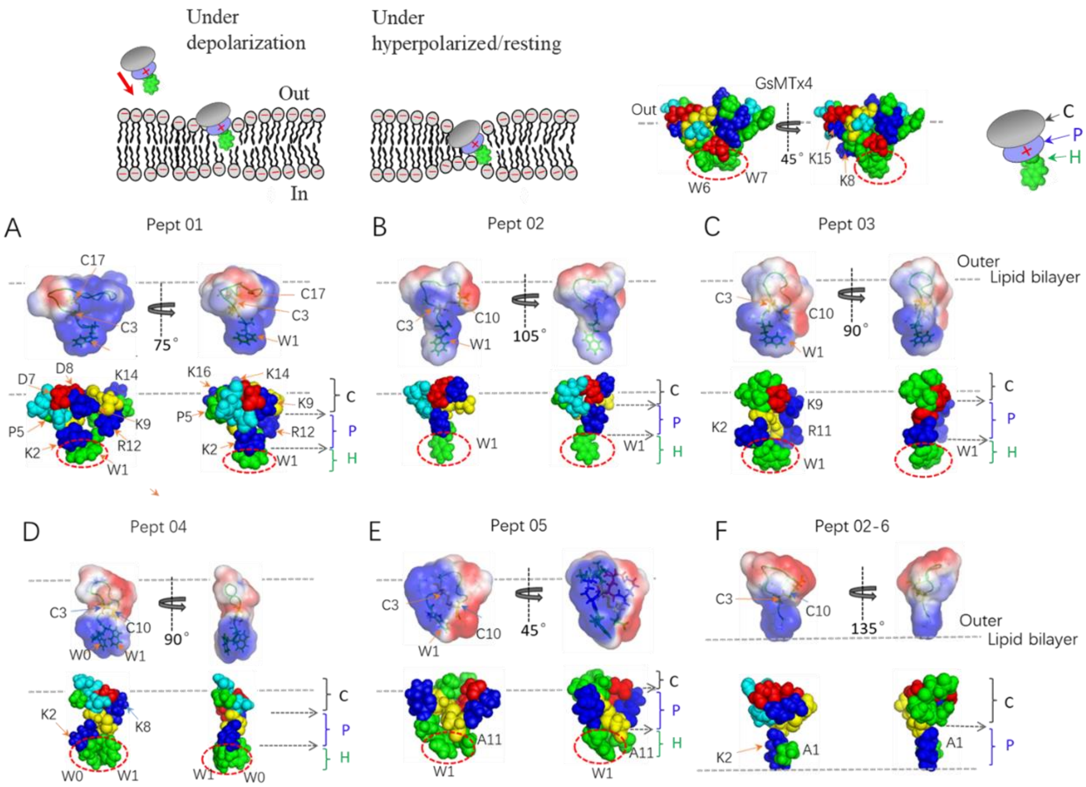

Figure S7. The structural comparisons for the synthetic peptides. (A) Pept 01. (B) Pept 02, (C) Pept 03. (D) Pept 04. (E) Pept 05 and (F) Pept 02-6. The structural models are represented as the surface (above) and space-filling (bellow) models. Above: the surface models. The peptide backbones are shown as green tubes, and the disulfide bonds formed with cystines by MD simulation are shown in yellow. Bellow: the space-filling models. The aromatic and hydrophobic residues are shown in green, positively charged residues are shown in blue, negatively charged residues in red, and cysteines in yellow. GsMTx4 (PDB: 1TYK) structures are shown for comparison (on the right above). Note, all synthetic short peptides identified to inhibit SAKcaC (e.g. Pepts 01, Pept 02, Pept 03, Pept 04, and Pept 05) have the common structural features: a hydrophobic head (indicated with H) formed with tyrosine (Try^0/Try^1 for Pept 04 and Trp^1 for others); one or more positively charged protrusion (Lys^2/R^{12} for Pept 01, Lys^2/Arg^{11} for Pept 03, and Lys^2 for others), and one pair of disulfide bond formed with cystines (highlighted in yellow in the backbone structures) which would act to sustain the spatial structures of the peptides. Structures are presented with the hydrophobic head down, which has been suggested to be involved in peptide partitioning into cell membrane for the parent peptide GsMTx4.

All structural models were obtained by using MD simulations under free production runs based on the structural model of Pept 01, which was built based on the solution structure of GsMTx4 (PDB code 1TYK). The insets above represent the two proposed modes for peptide actions under depolarized (left) *vs*. hyperpolarized/resting (middle) conditions following partitioning into the lipid bilayers.
